# Sequential Learning of Principal Curves: Summarizing Data Streams on the Fly

**DOI:** 10.3390/e23111534

**Published:** 2021-11-18

**Authors:** Le Li, Benjamin Guedj

**Affiliations:** 1 Department of Statistics, Central China Normal University, Wuhan 430079, China; leli@mail.ccnu.edu.cn; 2 Inria, Lille-Nord Europe Research Centre and Inria London, France and Centre for Artificial Intelligence, Department of Computer Science, University College London, London WC1V 6LJ, UK

**Keywords:** sequential learning, principal curves, data streams, regret bounds, greedy algorithm, sleeping experts

## Abstract

When confronted with massive data streams, summarizing data with dimension reduction methods such as PCA raises theoretical and algorithmic pitfalls. A principal curve acts as a nonlinear generalization of PCA, and the present paper proposes a novel algorithm to automatically and sequentially learn principal curves from data streams. We show that our procedure is supported by regret bounds with optimal sublinear remainder terms. A greedy local search implementation (called slpc, for sequential learning principal curves) that incorporates both sleeping experts and multi-armed bandit ingredients is presented, along with its regret computation and performance on synthetic and real-life data.

## 1. Introduction

**Figure 1:**
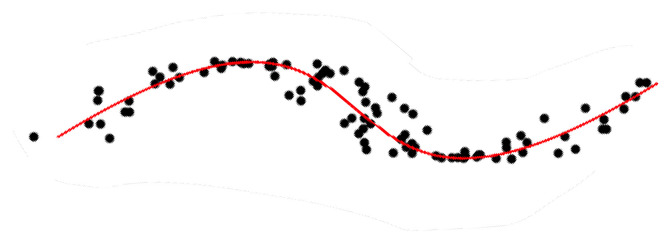
A principal curve.

Numerous methods have been proposed in the statistics and machine learning literature to sum up information and represent data by condensed and simpler-to-understand quantities. Among those methods, principal component analysis (PCA) aims at identifying the maximal variance axes of data. This serves as a way to represent data in a more compact fashion and hopefully reveal as well as possible their variability. PCA was introduced by [1,2] and further developed by [3]. This is one of the most widely used procedures in multivariate exploratory analysis targeting dimension reduction or feature extraction. Nonetheless, PCA is a linear procedure and the need for more sophisticated nonlinear techniques has led to the notion of principal curve. Principal curves may be seen as a nonlinear generalization of the first principal component. The goal is to obtain a curve which passes “in the middle” of data, as illustrated by Figure 1. This notion of skeletonization of data clouds has been at the heart of numerous applications in many different domains, such as physics [4,5], character and speech recognition [6,7], mapping and geology [5,8,9], to name but a few.

### 1.1. Earlier Works on Principal Curves

The original definition of principal curve dates back to [10]. A principal curve is a smooth ($C^{\infty }$) parameterized curve $f\left(s\right)=\left(f_{1}\left(s\right),\ldots ,f_{d}\left(s\right)\right)$ in $R^{d}$ which does not intersect itself, has finite length inside any bounded subset of $R^{d}$ and is self-consistent. This last requirement means that $f\left(s\right)=E[X|s_{f}\left(X\right)=s]$, where $X\in R^{d}$ is a random vector and the so-called projection index $s_{f}\left(x\right)$ is the largest real number *s* minimizing the squared Euclidean distance between f(s) and *x*, defined by

$$s_{f}\left(x\right)=\sup\left\{s:{∥x-f(s)∥}_{2}^{2}=\underset{\tau }{\inf}\;{∥x-f(\tau )∥}_{2}^{2}\right\}.$$

**Figure 2:**
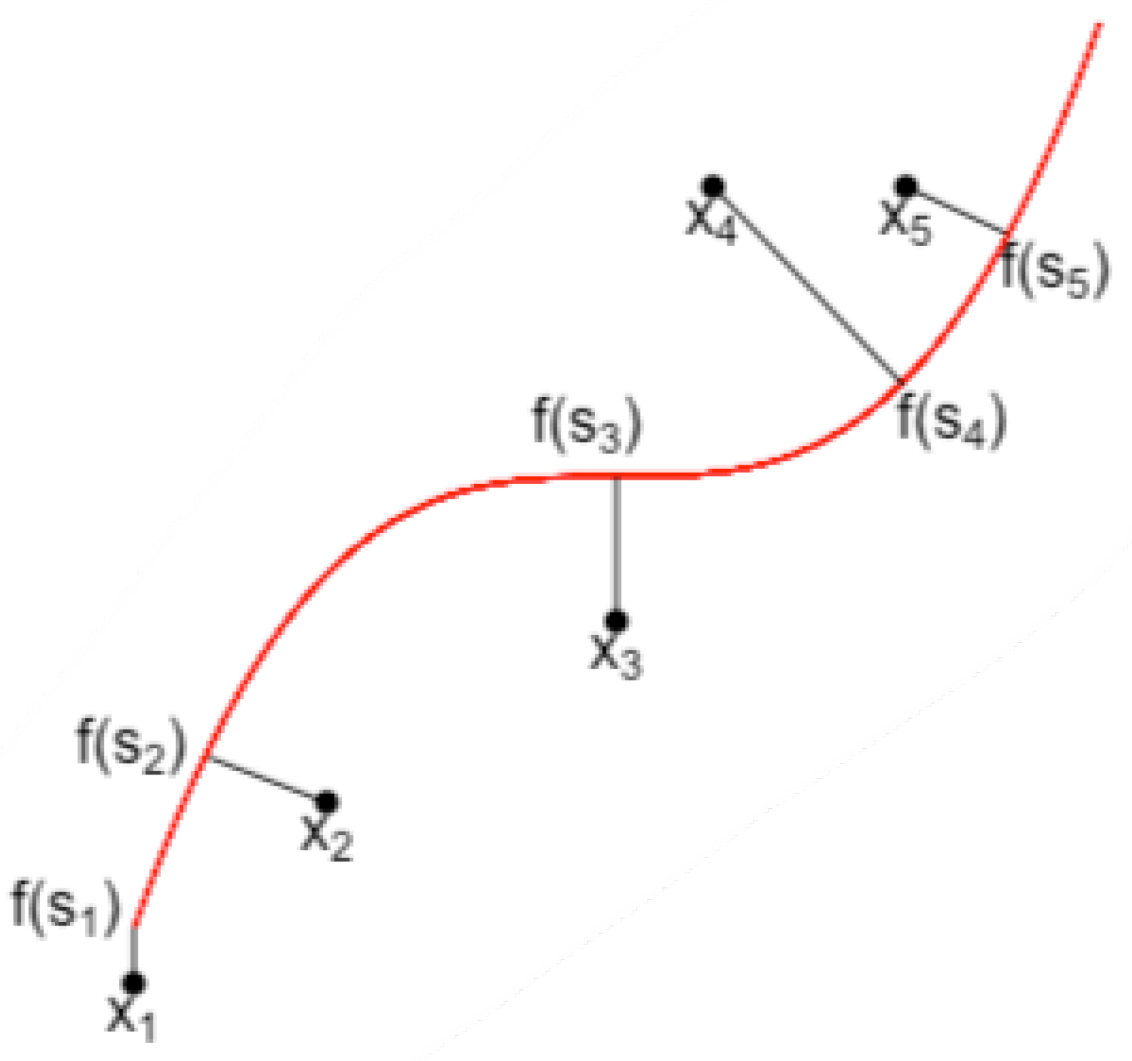
A principal curve and projections of data onto it.

Self-consistency means that each point of f is the average (under the distribution of *X*) of all data points projected on f, as illustrated by Figure 2.

However, an unfortunate consequence of this definition is that the existence is not guaranteed in general for a particular distribution, let alone for an online sequence for which no probabilistic assumption is made. In order to handle complex data structures, Ref. [11] proposed principal curves (PCOP) of principal oriented points (POPs) which are defined as the fixed points of an expectation function of points projected to a hyperplane minimising the total variance. To obtain POPs, a cluster analysis is performed on the hyperplane and only data in the local cluster are considered. Ref. [12] introduced the local principal curve (LPC), whose concept is similar to that of [11], but accelerates the computation of POPs by calculating local centers of mass instead of performing cluster analysis, and local principal component instead of principal direction. Later, Ref. [13] also considered LPC in data compression and regression to reduce the dimension of predictors space to low-dimension manifold. Ref. [14] extended the idea of localization to independent component analysis (ICA) by proposing a local-to-global non-linear ICA framework for visual and auditory signal. Ref. [15] considered principal curves from a different perspective: as the ridge of a smooth probability density function (PDF) generating dataset, where the ridges are collections of all points; the local gradient of a PDF is an eigenvector of the local Hessian, and the eigenvalues corresponding to the remaining eigenvectors are negative. To estimate principal curves based on this definition, the subspace constrained mean shift (SCMS) algorithm was proposed. All the local methods above require strong assumptions on the PDF, such as twice continuous differentiability, which may prove challenging to be satisfied in the settings of online sequential data. Ref. [16] proposed a new concept of principal curves which ensures its existence for a large class of distributions. Principal curves $f^{⋆}$ are defined as the curves minimizing the expected squared distance over a class $F_{L}$ of curves whose length is smaller than L>0; namely,

$$f^{⋆}\in \underset{f\in F_{L}}{\arg\inf}\;\Delta \left(f\right),$$

where

$$\Delta \left(f\right)=E\left[\Delta \left(f,X\right)\right]=E\left[\underset{s}{\inf}\;{∥f(s)-X∥}_{2}^{2}\right].$$

If ${E∥X∥}_{2}^{2}<\infty$, $f^{⋆}$ always exists but may not be unique. In practical situations where only i.i.d. copies $X_{1},\ldots ,X_{n}$ of *X* are observed, the method of [16] considers classes $F_{k,L}$ of all polygonal lines with *k* segments and length not exceeding *L*, and chooses an estimator ${\hat{f}}_{k,n}$ of $f^{⋆}$ as the one within $F_{k,L}$, which minimizes the empirical counterpart

$$\Delta_{n}\left(f\right)=\frac{1}{n}\sum_{i=1}^{n}\Delta \left(f,X_{i}\right)$$

of Δ(f). It is proved in [17] that if *X* is almost surely bounded and $k\propto n^{1/3}$, then

$$\Delta \left({\hat{f}}_{k,n}\right)-\Delta \left(f^{⋆}\right)=O\left(n^{-1/3}\right).$$

As the task of finding a polygonal line with *k* segments and length of at most *L* that minimizes $\Delta_{n}\left(f\right)$ is computationally costly, Ref. [17] proposed a polygonal line algorithm. This iterative algorithm proceeds by fitting a polygonal line with *k* segments and considerably speeds up the exploration part by resorting to gradient descent. The two steps (projection and optimization) are similar to what is done by the *k*-means algorithm. However, the polygonal line algorithm is not supported by theoretical bounds and leads to variable performance depending on the distribution of the observations.

**Figure 3:**
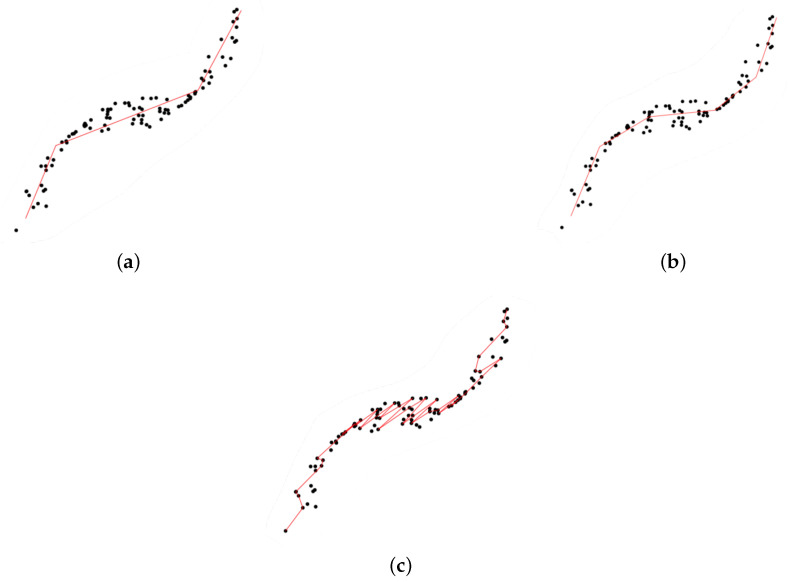
Principal curves with different numbers (*k*) of segments. (**a**) A too small *k*. (**b**) Right *k*. (**c**) A too large *k*.

As the number of segments, *k*, plays a crucial role (a too small a *k* value leads to a poor summary of data, whereas a too-large *k* yields overfitting; see Figure 3), Ref. [18] aimed to fill the gap by selecting an optimal *k* from both theoretical and practical perspectives.

Their approach relies strongly on the theory of model selection by penalization introduced by [19] and further developed by [20]. By considering countable classes ${\left\{F_{k,\ell }\right\}}_{k,\ell }$ of polygonal lines with *k* segments and total length ℓ≤L, and whose vertices are on a lattice, the optimal $\left(\hat{k},\hat{\ell }\right)$ is obtained as the minimizer of the criterion

$$\mathrm{crit}\left(k,\ell \right)=\Delta_{n}\left({\hat{f}}_{k,\ell }\right)+\mathrm{pen}\left(k,\ell \right),$$

where

$$\mathrm{pen}\left(k,\ell \right)=c_{0}\sqrt{\frac{k}{n}}+c_{1}\frac{\ell }{n}+c_{2}\frac{1}{\sqrt{n}}+\delta^{2}\sqrt{\frac{w_{k,\ell }}{2n}}$$

is a penalty function where δ stands for the diameter of observations and $w_{k,\ell }$ denotes the weight attached to class $F_{k,\ell }$; and it has constants $c_{0},c_{1},c_{2}$ depending on δ, maximum length *L* and a certain number of dimensions of observations. Ref.  [18] then proved that

(1) $$E\left[\Delta \left({\hat{f}}_{\hat{k},\hat{\ell }}\right)-\Delta \left(f^{⋆}\right)\right]\leq \underset{k,\ell }{\inf}\;\left\{E\left[\Delta \left({\hat{f}}_{k,\ell }\right)-\Delta \left(f^{⋆}\right)\right]+\mathrm{pen}\left(k,\ell \right)\right\}+\frac{\delta^{2}\Sigma }{2^{3/2}}\sqrt{\frac{\pi }{n}},$$

where Σ is a numerical constant. The expected loss of the final polygonal line ${\hat{f}}_{\hat{k},\hat{\ell }}$ is close to the minimal loss achievable over $F_{k,\ell }$ up to a remainder term decaying as $1/\sqrt{n}$.

### 1.2. Motivation

The big data paradigm—where collecting, storing and analyzing massive amounts of large and complex data becomes the new standard—commands one to revisit some of the classical statistical and machine learning techniques. The tremendous improvements of data acquisition infrastructures generates new continuous streams of data, rather than batch datasets. This has drawn great interest to sequential learning. Extending the notion of principal curves to the sequential settings opens up immediate practical application possibilities. As an example, path planning for passengers’ locations can help taxi companies to better optimize their fleet. Online algorithms that could yield instantaneous path summarization would be adapted to the sequential nature of geolocalized data. Existing theoretical works and practical implementations of principal curves are designed for the batch setting [7,16,17,18,21] and their adaptation to the sequential setting is not a smooth process. As an example, consider the algorithm in [18]. It is assumed that vertices of principal curves are located on a lattice, and its computational complexity is of order $O(nN^{p})$ where *n* is the number of observations, *N* the number of points on the lattice and *p* the maximum number of vertices. When *p* is large, running this algorithm at each epoch yields a monumental computational cost. In general, if data are not identically distributed or even adversary, algorithms that originally worked well in the batch setting may not be ideal when cast onto the online setting (see [22], Chapter 4). To the best of our knowledge, little effort has been put so far into extending principal curves algorithms to the sequential context.

Ref. [23] provided an incremental version of the SCMS algorithm [15] which is based on a definition of a principal curve as the ridge of a smooth probability density function generating observations. They applied the SCMS algorithm to the input points that are associated with the output points which are close to the new incoming sample and leave the remaining outputs unchanged. Hence, this algorithm can be used to deal with sequential data. As presented in the next section, our algorithm for sequentially updating principal curve vertices that are close to new data is similar in spirit to that of incremental SCMS. However, a difference is that our algorithm outputs polygonal lines. In addition, the computation complexity of our method is $O(n^{2})$, and incremental SCMS has $O(n^{3})$ complexity, where *n* is the number of observations. Ref. [24] considered sequential principal curves analysis in a fairly different setting in which the goal was to derive in an adaptive fashion a set of nonlinear sensors by using a set of preliminary principal curves. Unfolding sequentially principal curves and a sequential path for Jacobian integration were considered. The “sequential” in this setting represented the generalization of principal curves to principal surfaces or even a principal manifold of higher dimensions. This way of sequentially exploiting principal curves was firstly proposed by [11] and later extended by [14,25,26] to give curvilinear representations using sequence of local-to-global curves. In addition, Refs. [15,27,28] presented, respectively, principal polynomial and non-parametric regressions to capture the nonlinear nature of data. However, these methods are not originally designed for treating sequential data. The present paper aims at filling this gap: our goal was to propose an online perspective to principal curves by automatically and sequentially learning the best principal curve summarizing a data stream. Sequential learning takes advantage of the latest collected (set of) observations and therefore suffers a much smaller computational cost.

Sequential learning operates as follows: a blackbox reveals at each time *t* some deterministic value $x_{t},t=1,2,\ldots$, and a forecaster attempts to predict sequentially the next value based on past observations (and possibly other available information). The performance of the forecaster is no longer evaluated by its generalization error (as in the batch setting) but rather by a regret bound which quantifies the cumulative loss of a forecaster in the first *T* rounds with respect to some reference minimal loss. In sequential learning, the velocity of algorithms may be favored over statistical precision. An immediate use of aforecited techniques [17,18,21] at each time round *t* (treating data collected until *t* as a batch dataset) would result in a monumental algorithmic cost. Rather, we propose a novel algorithm which adapts to the sequential nature of data, i.e., which takes advantage of previous computations.

The contributions of the present paper are twofold. We first propose a sequential principal curve algorithm, for which we derived regret bounds. We then present an implementation, illustrated on a toy dataset and a real-life dataset (seismic data). The sketch of our algorithm’s procedure is as follows. At each time round *t*, the number of segments of $k_{t}$ is chosen automatically and the number of segments $k_{t+1}$ in the next round is obtained by only using information about $k_{t}$ and a small number of past observations. The core of our procedure relies on computing a quantity which is linked to the mode of the so-called Gibbs quasi-posterior and is inspired by quasi-Bayesian learning. The use of quasi-Bayesian estimators is especially advocated by the PAC-Bayesian theory, which originated in the machine learning community in the late 1990s, in the seminal works of [29] and McAllester [30,31]. The PAC-Bayesian theory has been successfully adapted to sequential learning problems; see, for example, Ref. [32] for online clustering. We refer to [33,34] for a recent overview of the field.

The paper is organized as follows. Section 2 presents our notation and our online principal curve algorithm, for which we provide regret bounds with sublinear remainder terms in Section 3. A practical implementation was proposed in Section 4, and we illustrate its performance on synthetic and real-life datasets in Section 5. Proofs of all original results claimed in the paper are collected in Section 6.

## 2. Notation

A parameterized curve in $R^{d}$ is a continuous function $f:I⟶R^{d}$ where I=[a,b] is a closed interval of the real line. The length of f is given by

$$L\left(f\right)=\lim_{M\to \infty }\;\left\{\underset{a=s_{0}<s_{1}<\cdots <s_{M}=b}{\sup}\;\;\sum_{i=1}^{M}{∥f\left(s_{i}\right)-f\left(s_{i-1}\right)∥}_{2}\right\}.$$

**Figure 4:**
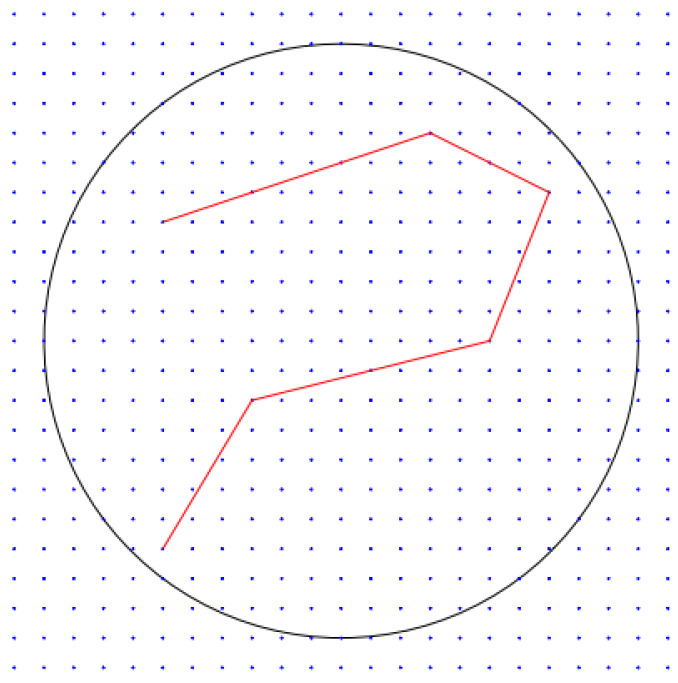
An example of a lattice $\Gamma_{\delta }$ in $R^{2}$ with δ=1 (spacing between blue points) and B(0,10) (black circle). The red polygonal line is composed of vertices in $Q_{\delta }=B\left(0,10\right)\;\cap \;\Gamma_{\delta }$.

Let $x_{1},x_{2},\ldots ,x_{T}\in B\left(0,\sqrt{d}R\right)\subset R^{d}$ be a sequence of data, where B(c,R) stands for the $\ell_{2}$-ball centered in $c\in R^{d}$ with radius R>0. Let $Q_{\delta }$ be a grid over $B(0,\sqrt{d}R)$, i.e., $Q_{\delta }=B\left(0,\sqrt{d}R\right)\cap \Gamma_{\delta }$ where $\Gamma_{\delta }$ is a lattice in $R^{d}$ with spacing δ>0. Let L>0 and define for each k∈⟦1,p⟧ the collection $F_{k,L}$ of polygonal lines f with *k* segments whose vertices are in $Q_{\delta }$ and such that L(f)≤L. Denote by $F_{p}=\cup_{k=1}^{p}F_{k,L}$ all polygonal lines with a number of segments ≤p, whose vertices are in $Q_{\delta }$ and whose length is at most *L*. Finally, let K(f) denote the number of segments of $f\in F_{p}$. This strategy is illustrated by Figure 4.

Our goal is to learn a time-dependent polygonal line which passes through the “middle” of data and gives a summary of all available observations $x_{1},\ldots ,x_{t-1}$ (denoted by ${\left(x_{s}\right)}_{1:(t-1)}$ hereafter) before time *t*. Our output at time *t* is a polygonal line ${\hat{f}}_{t}\in F_{p}$ depending on past information ${\left(x_{s}\right)}_{1:(t-1)}$ and past predictions ${\left({\hat{f}}_{s}\right)}_{1:(t-1)}$. When $x_{t}$ is revealed, the instantaneous loss at time *t* is computed as

(2) $$\Delta \left({\hat{f}}_{t},x_{t}\right)=\underset{s\in I}{\inf}\;{∥{\hat{f}}_{t}\left(s\right)-x_{t}∥}_{2}^{2}.$$

In what follows, we investigate regret bounds for the cumulative loss based on (2). Given a measurable space Θ (embedded with its Borel σ-algebra), we let P(Θ) denote the set of probability distributions on Θ, and for some reference measure π, we let $P_{\pi }\left(\Theta \right)$ be the set of probability distributions absolutely continuous with respect to π.

For any k∈⟦1,p⟧, let $\pi_{k}$ denote a probability distribution on $F_{k,L}$. We define the *prior*π on $F_{p}=\cup_{k=1}^{p}F_{k,L}$ as

$$\pi \left(f\right)=\sum_{k\in ⟦1,p⟧}w_{k}\pi_{k}\left(f\right){𝟙}_{\left\{f\in F_{k,L}\right\}},\;f\in F_{p},$$

where $w_{1},\ldots ,w_{p}\geq 0$ and $\sum_{k\in ⟦1,p⟧}w_{k}=1$.

We adopt a quasi-Bayesian-flavored procedure: consider the Gibbs quasi-posterior (note that this is not a proper posterior in all generality, hence the term “quasi”):

$${\hat{\rho }}_{t}\left(\cdot \right)\propto \exp\left(-\lambda S_{t}\left(\cdot \right)\right)\pi \left(\cdot \right),$$

where

$$S_{t}\left(f\right)=S_{t-1}\left(f\right)+\Delta \left(f,x_{t}\right)+\frac{\lambda }{2}{\left(\Delta \left(f,x_{t}\right)-\Delta \left({\hat{f}}_{t},x_{t}\right)\right)}^{2},$$

as advocated by [32,35] who then considered realizations from this quasi-posterior. In the present paper, we will rather focus on a quantity linked to the mode of this quasi-posterior. Indeed, the mode of the quasi-posterior ${\hat{\rho }}_{t+1}$ is

$$\arg\min_{f\in F_{p}}\left\{\underset{\left(i\right)}{\underbrace{\sum_{s=1}^{t}\Delta \left(f,x_{s}\right)}}+\underset{\left(ii\right)}{\underbrace{\frac{\lambda }{2}\sum_{s=1}^{t}{\left(\Delta \left(f,x_{t}\right)-\Delta \left({\hat{f}}_{t},x_{t}\right)\right)}^{2}}}+\underset{\left(iii\right)}{\underbrace{\frac{\ln\pi (f)}{\lambda }}}\right\},$$

where *(i)* is a cumulative loss term, *(ii)* is a term controlling the variance of the prediction f to past predictions ${\hat{f}}_{s},s\leq t$, and *(iii)* can be regarded as a penalty function on the complexity of f if π is well chosen. This mode hence has a similar flavor to follow the best expert or follow the perturbed leader in the setting of prediction with experts (see [22,36], Chapters 3 and 4) if we consider each $f\in F_{p}$ as an expert which always delivers constant advice. These remarks yield Algorithm 1.
**Algorithm 1** Sequentially learning principal curves.   1:**Input parameters**: $p>0,\eta >0,\pi \left(z\right)=e^{-z}1_{\left\{z>0\right\}}$ and penalty function $h:F_{p}\to R^{+}$   2:**Initialization**: For each $f\in F_{p}$, draw $z_{f}∼\pi$ and $\Delta_{f,0}=\frac{1}{\eta }\left(h\left(f\right)-z_{f}\right)$   3:**For t=1,…,T**   4:      Get the data $x_{t}$   5:      Obtain
$${\hat{f}}_{t}=\underset{f\in F_{p}}{\arg\inf}\left\{\sum_{s=0}^{t-1}\Delta_{f,s}\right\},$$      where $\Delta_{f,s}=\Delta \left(f,x_{s}\right)$, s≥1.   6:**End for**

## 3. Regret Bounds for Sequential Learning of Principal Curves

We now present our main theoretical results.

**Theorem** **1.** *For any sequence ${\left(x_{t}\right)}_{1:T}\in B\left(0,\sqrt{d}R\right)$, R≥0 and any penalty function $h:F_{p}\to R^{+}$, let $\pi \left(z\right)=e^{-z}1_{\left\{z>0\right\}}$. Let $0<\eta \leq \frac{1}{d{\left(2R+\delta \right)}^{2}}$; then the procedure described in Algorithm 1 satisfies*

$$\sum_{t=1}^{T}E_{\pi }\left[\Delta ({\hat{f}}_{t},x_{t})\right]\leq \left(1+c_{0}\left(e-1\right)\eta \right)S_{T,h,\eta }+\frac{1}{\eta }\left(1+\ln\sum_{f\in F_{p}}e^{-h(f)}\right),$$

*where $c_{0}=d{\left(2R+\delta \right)}^{2}$ and*

$$S_{T,h,\eta }=\underset{k\in ⟦1,p⟧}{\inf}\left\{\underset{\begin{array}{c} f\in F_{p}\\ K(f)=k \end{array}}{\inf}\left\{\sum_{t=1}^{T}\Delta \left(f,x_{t}\right)+\frac{h(f)}{\eta }\right\}\right\}.$$

The expectation of the cumulative loss of polygonal lines ${\hat{f}}_{1},\ldots ,{\hat{f}}_{T}$ is upper-bounded by the smallest penalized cumulative loss over all k∈{1,…,p} up to a multiplicative term $\left(1+c_{0}\left(e-1\right)\eta \right)$, which can be made arbitrarily close to 1 by choosing a small enough η. However, this will lead to both a large h(f)/η in $S_{T,h,\eta }$ and a large $\frac{1}{\eta }\left(1+\ln\sum_{f\in F_{p}}e^{-h(f)}\right)$. In addition, another important issue is the choice of the penalty function *h*. For each $f\in F_{p}$, h(f) should be large enough to ensure a small $\sum_{f\in F_{p}}e^{-h(f)}$, but not too large to avoid overpenalization and a larger value for $S_{T,h,\eta }$. We therefore set

(3) $$h\left(f\right)\geq \ln\left(pe\right)+\ln|\{f\in F_{p},K\left(f\right)=k\}|$$

for each f with *k* segments (where |M| denotes the cardinality of a set *M*) since it leads to

$$\sum_{f\in F_{p}}e^{-h(f)}=\sum_{k\in ⟦1,p⟧}\sum_{\begin{array}{c} f\in F_{p}\\ K(f)=k \end{array}}e^{-h(f)}\leq \sum_{k\in ⟦1,p⟧}\frac{1}{pe}\leq \frac{1}{e}.$$

The penalty function $h\left(f\right)=c_{1}K\left(f\right)+c_{2}L+c_{3}$ satisfies (3), where $c_{1},c_{2},c_{3}$ are constants depending on *R*, *d*, δ, *p* (this is proven in Lemma 3, in Section 6). We therefore obtain the following corollary.

**Corollary** **1.** *Under the assumptions of Theorem 1, let*

$$\eta =\min\left\{\frac{1}{d{\left(2R+\delta \right)}^{2}},\sqrt{\frac{c_{1}p+c_{2}L+c_{3}}{c_{0}\left(e-1\right)\inf_{f\in F_{p}}\sum_{t=1}^{T}\Delta \left(f,x_{t}\right)}}\right\}.$$

*Then*

$$\begin{array}{c} \sum_{t=1}^{T}E\left[\Delta ({\hat{f}}_{t},x_{t})\right]\leq \underset{k\in ⟦1,p⟧}{\inf}\left\{\underset{\begin{array}{c} f\in F_{p}\\ K(f)=k \end{array}}{\inf}\left\{\sum_{t=1}^{T}\Delta \left(f,x_{t}\right)+\sqrt{c_{0}\left(e-1\right)r_{T,k,L}}\right\}\right\}\\ \;\;\;\;\;\;\;\;\;\;\;\;\;\;\;+\sqrt{c_{0}\left(e-1\right)r_{T,p,L}}+c_{0}\left(e-1\right)\left(c_{1}p+c_{2}L+c_{3}\right), \end{array}$$

*where $r_{T,k,L}=\inf_{f\in F_{p}}\sum_{t=1}^{T}\Delta \left(f,x_{t}\right)\left(c_{1}k+c_{2}L+c_{3}\right)$.*

**Proof.**  Note that

$$\sum_{t=1}^{T}E\left[\Delta ({\hat{f}}_{t},x_{t})\right]\leq S_{T,h,\eta }+\eta c_{0}\left(e-1\right)\underset{f\in F_{p}}{\inf}\sum_{t=1}^{T}\Delta \left(f,x_{t}\right)+c_{0}\left(e-1\right)\left(c_{0}p+c_{2}L+c_{3}\right),$$

and we conclude by setting

$$\eta =\sqrt{\frac{c_{1}p+c_{2}L+c_{3}}{c_{0}\left(e-1\right)\inf_{f\in F_{p}}\sum_{t=1}^{T}\Delta \left(f,x_{t}\right)}}.$$

 □

Sadly, Corollary 1 is not of much practical use since the optimal value for η depends on $\inf_{f\in F_{p}}\sum_{t=1}^{T}\Delta \left(f,x_{t}\right)$ which is obviously unknown, even more so at time t=0. We therefore provide an adaptive refinement of Algorithm 1 in the following Algorithm 2.
**Algorithm 2** Sequentially and adaptively learning principal curves.   1:**Input parameters**: p>0, L>0, π, *h* and $\eta_{0}=\frac{\sqrt{c_{1}p+c_{2}L+c_{3}}}{c_{0}\sqrt{e-1}}$   2:**Initialization**: For each $f\in F_{p}$, draw $z_{f}∼\pi$, $\Delta_{f,0}=\frac{1}{\eta_{0}}\left(h\left(f\right)-z_{f}\right)$ and ${\hat{f}}_{0}=\underset{f\in F_{p}}{\arg\inf}\;\Delta_{f,0}$   3:**For t=1,…,T**   4:      Compute $\eta_{t}=\frac{\sqrt{c_{1}p+c_{2}L+c_{3}}}{c_{0}\sqrt{(e-1)t}}$   5:      Get data $x_{t}$ and compute $\Delta_{f,t}=\Delta \left(f,x_{t}\right)+\left(\frac{1}{\eta_{t}}-\frac{1}{\eta_{t-1}}\right)\left(h\left(f\right)-z_{f}\right)$   6:      Obtain
(4)$${\hat{f}}_{t}=\underset{f\in F_{p}}{\arg\inf}\;\left\{\sum_{s=0}^{t-1}\Delta_{f,s}\right\}.$$   7:**End for**

**Theorem** **2.** *For any sequence ${\left(x_{t}\right)}_{1:T}\in B\left(0,\sqrt{d}R\right),R\geq 0$, let $h\left(f\right)=c_{1}K\left(f\right)+c_{2}L+c_{3}$ where $c_{1}$, $c_{2}$, $c_{3}$ are constants depending on R,d,δ,lnp. Let $\pi \left(z\right)=e^{-z}1_{\left\{z>0\right\}}$ and*

$$\eta_{0}=\frac{\sqrt{c_{1}p+c_{2}L+c_{3}}}{c_{0}\sqrt{e-1}},\;\eta_{t}=\frac{\sqrt{c_{1}p+c_{2}L+c_{3}}}{c_{0}\sqrt{(e-1)t}},$$

*where t≥1 and $c_{0}=d{\left(2R+\delta \right)}^{2}$. Then the procedure described in Algorithm 2 satisfies*

$$\begin{array}{c} \sum_{t=1}^{T}E\left[\Delta ({\hat{f}}_{t},x_{t})\right]\leq \underset{k\in ⟦1,p⟧}{\inf}\left\{\underset{\begin{array}{c} f\in F_{p}\\ K(f)=k \end{array}}{\inf}\left\{\sum_{t=1}^{T}\Delta \left(f,x_{t}\right)+c_{0}\sqrt{\left(e-1\right)T\left(c_{1}k+c_{2}L+c_{3}\right)}\right\}\right\}\\ \;\;\;\;\;\;\;\;\;\;\;\;\;\;\;\;\;\;\;\;+2c_{0}\sqrt{\left(e-1\right)T\left(c_{1}p+c_{2}L+c_{3}\right)}. \end{array}$$

The message of this regret bound is that the expected cumulative loss of polygonal lines ${\hat{f}}_{1},\ldots ,{\hat{f}}_{T}$ is upper-bounded by the minimal cumulative loss over all k∈{1,…,p}, up to an additive term which is sublinear in *T*. The actual magnitude of this remainder term is $\sqrt{kT}$. When *L* is fixed, the number *k* of segments is a measure of complexity of the retained polygonal line. This bound therefore yields the same magnitude as (1), which is the most refined bound in the literature so far ([18] where the optimal values for *k* and *L* were obtained in a model selection fashion).

## 4. Implementation

The argument of the infimum in Algorithm 2 is taken over $F_{p}=\cup_{k=1}^{p}F_{k,L}$ which has a cardinality of order ${\left|Q_{\delta }\right|}^{p}$, making any greedy search largely time-consuming. We instead turn to the following strategy: Given a polygonal line ${\hat{f}}_{t}\in F_{k_{t},L}$ with $k_{t}$ segments, we consider, with a certain proportion, the availability of ${\hat{f}}_{t+1}$ within a neighborhood $U({\hat{f}}_{t})$ (see the formal definition below) of ${\hat{f}}_{t}$. This consideration is well suited for the principal curves setting, since if observation $x_{t}$ is close to ${\hat{f}}_{t}$, one can expect that the polygonal line which well fits observations $x_{s},s=1,\ldots ,t$ lies in a neighborhood of ${\hat{f}}_{t}$. In addition, if each polygonal line f is regarded as an action, we no longer assume that all actions are available at all times, and allow the set of available actions to vary at each time. This is a model known as “sleeping experts (or actions)” in prior work [37,38]. In this setting, defining the regret with respect to the best action in the whole set of actions in hindsight remains difficult, since that action might sometimes be unavailable. Hence, it is natural to define the regret with respect to the best ranking of all actions in the hindsight according to their losses or rewards, and at each round one chooses among the available actions by selecting the one which ranks the highest. Ref. [38] introduced this notion of regret and studied both the full-information (best action) and partial-information (multi-armed bandit) settings with stochastic and adversarial rewards and adversarial action availability. They pointed out that the **EXP4** algorithm [37] attains the optimal regret in the adversarial rewards case but has a runtime exponential in the number of all actions. Ref. [39] considered full and partial information with stochastic action availability and proposed an algorithm that runs in polynomial time. In what follows, we materialize our implementation by resorting to “sleeping experts”, i.e., a special set of available actions that adapts to the setting of principal curves.

Let σ denote an ordering of $|F_{p}|$ actions, and $A_{t}$ a subset of the available actions at round *t*. We let $\sigma (A_{t})$ denote the highest ranked action in $A_{t}$. In addition, for any action $f\in F_{p}$ we define the reward $r_{f,t}$ of f at round t,t≥0 by

$$r_{f,t}=c_{0}-\Delta \left(f,x_{t}\right).$$

It is clear that $r_{f,t}\in \left(0,c_{0}\right)$. The convention from losses to gains is done in order to facilitate the subsequent performance analysis. The reward of an ordering σ is the cumulative reward of the selected action at each time:

$$\sum_{t=1}^{T}r_{\sigma (A_{t}),t},$$

and the reward of the best ordering is $\max_{\sigma }\sum_{t=0}^{T}r_{\sigma (A_{t}),t}$ (respectively, $E\left[\max_{\sigma }\sum_{t=1}^{T}r_{\sigma (A_{t}),t}\right]$ when $A_{t}$ is stochastic).

Our procedure starts with a **partition** step which aims at identifying the “relevant” neighborhood of an observation $x\in R^{d}$ with respect to a given polygonal line, and then proceeds with the definition of the **neighborhood** of an action f. We then provide the full implementation and prove a regret bound.

**Figure 5:**
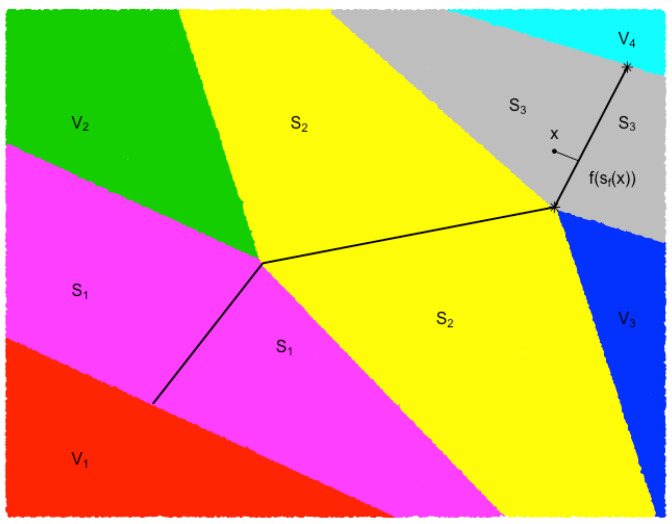
An example of a Voronoi partition.

**Partition.** For any polygonal line f with *k* segments, we denote by $\vec{V}=\left(v_{1},\ldots ,v_{k+1}\right)$ its vertices and by $s_{i},i=1,\ldots ,k$ the line segments connecting $v_{i}$ and $v_{i+1}$. In the sequel, we use $f(\vec{V})$ to represent the polygonal line formed by connecting consecutive vertices in $\vec{V}$ if no confusion arises. Let $V_{i},i=1,\ldots ,k+1$ and $S_{i},i=1,\ldots ,k$ be the Voronoi partitions of $R^{d}$ with respect to f, i.e., regions consisting of all points closer to vertex $v_{i}$ or segment $s_{i}$. Figure 5 shows an example of Voronoi partition with respect to f with three segments.

**Neighborhood.** For any $x\in R^{d}$, we define the neighborhood N(x) with respect to f as the union of all Voronoi partitions whose closure intersects with two vertices connecting the projection $f(s_{f}\left(x\right))$ of *x* to f. For example, for the point *x* in Figure 5, its neighborhood N(x) is the union of $S_{2},V_{3},S_{3}$ and $V_{4}$. In addition, let $N_{t}\left(x\right)=\left\{x_{s}\in N\left(x\right),s=1,\ldots ,t.\right\}$ be the set of observations $x_{1:t}$ belonging to $N\left(x\right)$ and ${\bar{N}}_{t}\left(x\right)$ be its average. Let $D\left(M\right)=\sup_{x,y\in M}||x-{y||}_{2}$ denote the diameter of set $M\subset R^{d}$. We finally define the local grid $Q_{\delta ,t}\left(x\right)$ of $x\in R^{d}$ at time *t* as

$$Q_{\delta ,t}\left(x\right)=B\left({\bar{N}}_{t}\left(x\right),D\left(N_{t}(x\right)\right)\cap Q_{\delta }.$$

We can finally proceed to the definition of the neighborhood $U({\hat{f}}_{t})$ of ${\hat{f}}_{t}$. Assume ${\hat{f}}_{t}$ has $k_{t}+1$ vertices $\vec{V}=\left(\underset{\left(i\right)}{\underbrace{v_{1:i_{t}-1}}},\underset{\left(ii\right)}{\underbrace{v_{i_{t}:j_{t}-1}}},\underset{\left(iii\right)}{\underbrace{v_{j_{t}:k_{t}+1}}}\right)$, where vertices of (ii) belong to $Q_{\delta ,t}\left(x_{t}\right)$ while those of (i) and (iii) do not. The neighborhood $U({\hat{f}}_{t})$ consists of f sharing vertices (i) and (iii) with ${\hat{f}}_{t}$, but can be equipped with different vertices (ii) in $Q_{\delta ,t}\left(x_{t}\right)$; i.e.,

$$U\left({\hat{f}}_{t}\right)=\left\{f\left(\vec{V}\right),\;\vec{V}=\left(v_{1:i_{t}-1},v_{1:m},v_{j_{t}:k_{t}+1}\right)\right\},$$

where $v_{1:m}\in Q_{\delta ,t}\left(x_{t}\right)$ and *m* is given by

$$m=\left\{\begin{array}{cc} j_{t}-i_{t}-1 & \mathrm{reduce segments by 1 unit},\\ j_{t}-i_{t} & \mathrm{same number of segments},\\ j_{t}-i_{t}+1 & \mathrm{increase segments by 1 unit}. \end{array}\right.$$

In Algorithm 3, we initiate the principal curve ${\hat{f}}_{1}$ as the first component line segment whose vertices are the two farthest projections of data $x_{1:t_{0}}$ ($t_{0}$ can be set to 20 in practice) on the first component line. The reward of f at round *t* in this setting is therefore $r_{f,t}=c_{0}-\Delta \left(f,x_{t_{0}+t}\right)$. Algorithm 3 has an exploration phase (when $I_{t}=1$) and an exploitation phase ($I_{t}=0$). In the exploration phase, it is allowed to observe rewards of all actions and to choose an optimal perturbed action from the set $F_{p}$ of all actions. In the exploitation phase, only rewards of a part of actions can be accessed and rewards of others are estimated by a constant, and we update our action from the neighborhood $U\left({\hat{f}}_{t-1}\right)$ of the previous action ${\hat{f}}_{t-1}$. This local update (or search) greatly reduces computation complexity since $|U\left({\hat{f}}_{t-1}\right)|\ll \left|F_{p}\right|$ when *p* is large. In addition, this local search will be enough to account for the case when $x_{t}$ locates in $U\left({\hat{f}}_{t-1}\right)$. The parameter β needs to be carefully calibrated since it should not be too large to ensure that the condition cond(t) is non-empty; otherwise, all rewards are estimated by the same constant and thus lead to the same descending ordering of tuples for both $\left(\sum_{s=1}^{t-1}{\hat{r}}_{f,s},f\in F_{p}\right)$ and $\left(\sum_{s=1}^{t}{\hat{r}}_{f,s},f\in F_{p}\right)$. Therefore, we may face the risk of having ${\hat{f}}_{t+1}$ in the neighborhood of ${\hat{f}}_{t}$ even if we are in the exploration phase at time t+1. Conversely, very small β could result in large bias for the estimation $\frac{r_{f,t}}{P\left({\hat{f}}_{t}=f|H_{t}\right)}$ of $r_{f,t}$. Note that the exploitation phase is close yet different to the label efficient prediction ([40], Remark 1.1) since we allow an action at time *t* to be different from the previous one. Ref. [41] proposed the *geometric resampling* method to estimate the conditional probability $P\left({\hat{f}}_{t}=f|H_{t}\right)$ since this quantity often does not have an explicit form. However, due to the simple exponential distribution of $z_{f}$ chosen in our case, an explicit form of $P\left({\hat{f}}_{t}=f|H_{t}\right)$ is straightforward.
**Algorithm 3** A locally greedy algorithm for sequentially learning principal curves.   1:**Input parameters**: p>0, R>0, L>0, ϵ>0, α>0, 1>β>0 and any penalty function *h*   2:**Initialization**: Given ${\left(x_{t}\right)}_{1:t_{0}}$, obtain ${\hat{f}}_{1}$ as the first principal component   3:**For t=2,…,T**   4:      Draw $I_{t}∼Bernoulli\left(\epsilon \right)$ and $z_{f}∼\pi$.   5:      Let
$${\hat{\sigma }}_{t}=\mathrm{sort}\left(f,\;\sum_{s=1}^{t-1}{\hat{r}}_{f,s}-\frac{1}{\eta_{t-1}}h\left(f\right)+\frac{1}{\eta_{t-1}}z_{f}\right),$$
i.e., sorting all $f\in F_{p}$ in descending order according to their perturbed cumulative reward till t−1.   6:      If $I_{t}=1$, set $A_{t}=F_{p}$ and ${\hat{f}}_{t}={\hat{\sigma }}^{t}\left(A_{t}\right)$ and observe $r_{{\hat{f}}_{t},t}$   7:            
$${\hat{r}}_{f,t}=r_{f,t}\;\mathrm{for}\;f\in F_{p}.$$   8: 
     If $I_{t}=0$, set $A_{t}=U\left({\hat{f}}_{t-1}\right)$, ${\hat{f}}_{t}={\hat{\sigma }}^{t}\left(A_{t}\right)$ and observe $r_{{\hat{f}}_{t},t}$   9:            
$${\hat{r}}_{f,t}=\left\{\begin{array}{ccc} \frac{r_{f,t}}{P\left({\hat{f}}_{t}=f|H_{t}\right)} & if\;f\in U\left({\hat{f}}_{t-1}\right)\cap cond\left(t\right)\;\mathrm{and}\;{\hat{f}}_{t}=f,\alpha & \mathrm{otherwise}, \end{array}\right.$$
where $H_{t}$ denotes all the randomness before time *t* and $\mathrm{cond}\left(t\right)=\left\{f\in F_{p}:P\left({\hat{f}}_{t}=f|H_{t}\right)>\beta \right\}$. In particular, when t=1, we set ${\hat{r}}_{f,1}=r_{f,1}$ for all $f\in F_{p}$, $U\left({\hat{f}}_{0}\right)=\emptyset$ and ${\hat{r}}_{{\hat{\sigma }}^{1}\left(U\left({\hat{f}}_{0}\right)\right),1}\equiv 0$.   10:**End for**

**Theorem** **3.** *Assume that p>6, $T\geq 2|F_{p}{|}^{2}$ and let $\beta ={\left|F_{p}\right|}^{-\frac{1}{2}}T^{-\frac{1}{4}}$, $\alpha =\frac{c_{0}}{\beta }$, ${\hat{c}}_{0}=\frac{2c_{0}}{\beta }$, $\epsilon =1-{\left|F_{p}\right|}^{\frac{1}{2}-\frac{3}{p}}T^{-\frac{1}{4}}$ and*

$$\eta_{1}=\eta_{2}=\cdots =\eta_{T}=\frac{\sqrt{c_{1}p+c_{2}L+c_{3}}}{\sqrt{T(e-1)}{\hat{c}}_{0}}.$$

*Then the procedure described in Algorithm 3 satisfies the regret bound*

$$\sum_{t=1}^{T}E\left[\Delta \left({\hat{f}}_{t},x_{t}\right)\right]\leq \underset{f\in F_{p}}{\inf}E\left[\sum_{t=1}^{T}\Delta \left(f,x_{t}\right)\right]+O\left(T^{\frac{3}{4}}\right).$$

The proof of Theorem 3 is presented in Section 6. The regret is upper bounded by a term of order $\left({\left|F_{p}\right|}^{\frac{1}{2}}T^{\frac{3}{4}}\right)$, sublinear in *T*. The term $\left(1-\epsilon \right)c_{0}T=c_{0}{\left|F_{p}\right|}^{\frac{1}{2}}T^{\frac{3}{4}}$ is the price to pay for the local search (with a proportion 1−ϵ) of polygonal line ${\hat{f}}_{t}$ in the neighborhood of the previous ${\hat{f}}_{t-1}$. If ϵ=1, we would have that ${\hat{c}}_{0}=c_{0}$, and the last two terms in the first inequality of Theorem 3 would vanish; hence, the upper bound reduces to Theorem 2. In addition, our algorithm achieves an order that is smaller (from the perspective of both the number $\left|F_{p}\right|$ of all actions and the total rounds *T*) than [39] since at each time, the availability of actions for our algorithm can be either the whole action set or a neighborhood of the previous action while [39] consider at each time only partial and independent stochastic available set of actions generated from a predefined distribution.

## 5. Numerical Experiments

We illustrate the performance of Algorithm 3 on synthetic and real-life data. Our implementation (hereafter denoted by slpc—Sequential Learning of Principal Curves) is conducted with the R language and thus our most natural competitors are the R package princurve, which is the algorithm from [10], and incremental, which is the algorithm from SCMS [23]. We let p=50, $R=\max_{t=1,\ldots ,T}{\left||x|\right|}_{2}/\sqrt{d}$, $L=0.1p\sqrt{d}R$. The spacing δ of the lattice is adjusted with respect to data scale.

**Synthetic data** We generate a dataset $\left\{x_{t}\in R^{2},t=1,\ldots ,500\right\}$ uniformly along the curve $y=0.05\times {\left(x-5\right)}^{3}$, x∈[0,10]. Table 1 shows the regret (first row) for

- the ground truth (sum of squared distances of all points to the true curve),
- princurve and incremental SCMS (sum of squared distances between observation $x_{t+1}$ and fitted princurve on observations $x_{1:t}$),
- slpc (regret being equal to $\sum_{t=0}^{T-1}E\left[\Delta \left({\hat{f}}_{t+1},x_{t+1}\right)\right]$ in both cases).

The mean computation time with different values for the time horizons *T* are also reported.

**Figure 6:**
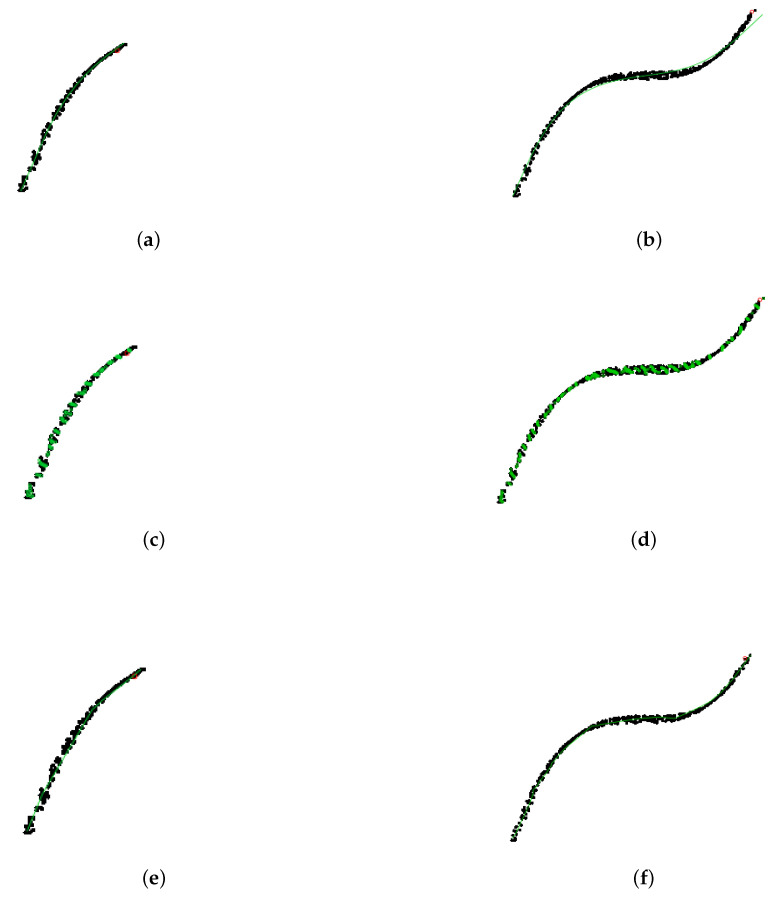
Synthetic data. Black dots represent data $x_{1:t}$. The red point is the new observation $x_{t+1}$. princurve (solid red) and slpc (solid green). (**a**) t=150, princurve. (**b**) t=450, princurve. (**c**) t=150, incremental SCMS. (**d**) t=450, incremental SCMS. (**e**) t=150, slpc. (**f**) t=450, slpc.

**Figure 7:**
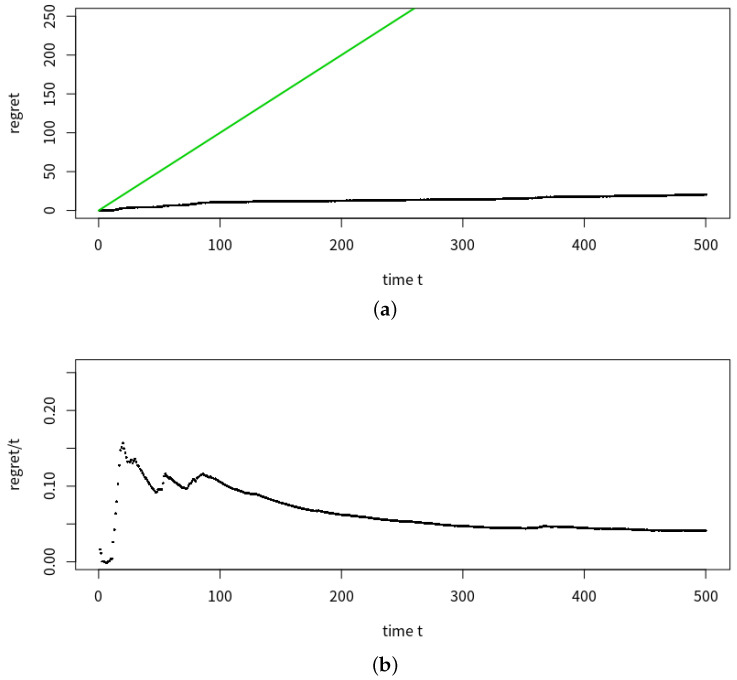
Mean estimation of regret and per-round regret of slpc with respect to time round *t*, for the horizon T=500. (**a**) Mean estimation of the regret of slpc over 20 trials (black line) and a bisection line (green) with respect to time round *t*. (**b**) Per-round of estimated regret of slpc with respect to *t*.

Table 1 demonstrates the advantages of our method slpc, as it achieved the optimal tradeoff between performance (in terms of regret) and runtime. Although princurve outperformed the other two algorithms in terms of computation time, it yielded the largest regret, since it outputs a curve which does not pass in “the middle of data” but rather bends towards the curvature of the data cloud, as shown in Figure 6 where the predicted principal curves ${\hat{f}}_{t+1}$ for princurve, incremental SCMS and slpc are presented. incremental SCMS and slpc both yielded satisfactory results, although the mean computation time of splc was significantly smaller than that of incremental SCMS (the reason being that eigenvectors of the Hessian of PDF need to be computed in incremental SCMS). Figure 7 showed, respectively, the estimation of the regret of slpc and its per-round value (i.e., the cumulative loss divided by the number of rounds) both with respect to the round *t*. The jumps in the per-round curve occurred at the beginning, due to the initialization from a first principal component and to the collection of new data. When data accumulates, the vanishing pattern of the per-round curve illustrates that the regret is sublinear in *t*, which matches our aforementioned theoretical results.

**Figure 8:**
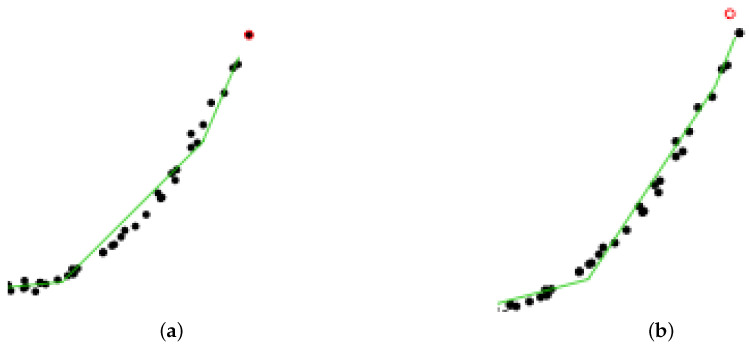
Synthetic data. Zooming in: how a new data point impacts the principal curve only locally. (**a**) At time t=97. (**b**) And at time t=98.

In addition, to better illustrate the way slpc works between two epochs, Figure 8 focuses on the impact of collecting a new data point on the principal curve. We see that only a local vertex is impacted, whereas the rest of the principal curve remains unaltered. This cutdown in algorithmic complexity is one the key assets of slpc.

**Synthetic data in high dimension.** We also apply our algorithm on a dataset $\{x_{t}\in R^{6},$t=1,2,…,200} in higher dimension. It is generated uniformly along a parametric curve whose coordinates are

$$\left(\begin{array}{c} 0.5t\cos(t)\\ 0.5t\sin(t)\\ 0.5t\\ -t\\ \sqrt{t}\\ 2\ln(t+1) \end{array}\right)$$

where *t* takes 100 equidistant values in [0,2π]. To the best of our knowledge, [10,16,18] only tested their algorithm on 2-dimensional data. This example aims at illustrating that our algorithm also works on higher dimensional data. Table 2 shows the regret for the ground truth, princurve and slpc.

**Figure 9:**
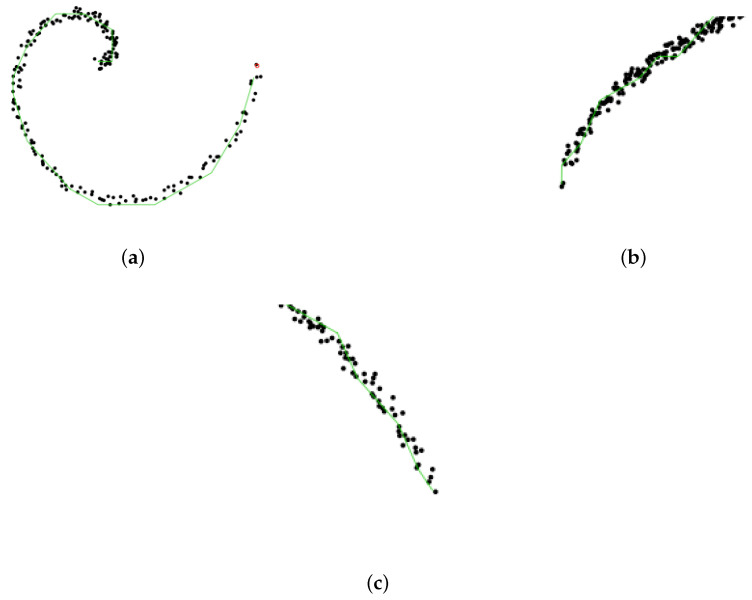
slpc (green line) on synthetic high dimensional data from different perspectives. Black dots represent recordings $x_{1:99}$; the red dot is the new recording $x_{200}$. (**a**) slpc, t=199, 1st and 2nd coordinates. (**b**) slpc, t=199, 3th and 5th coordinates. (**c**) slpc, t=199, 4th and 6th coordinates.

In addition, Figure 9 shows the behaviour of slpc (green) on each dimension.

**Figure 10:**
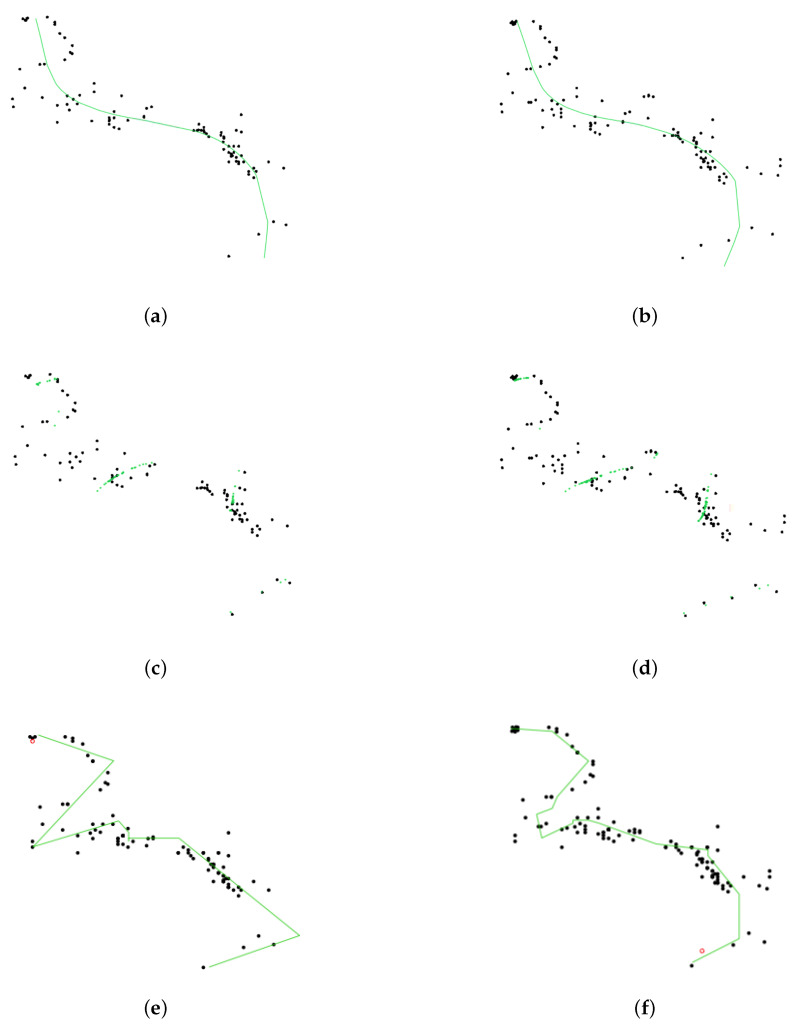
Seismic data. Black dots represent seismic recordings $x_{1:t}$; the red dot is the new recording $x_{t+1}$. (**a**) princurve, t=100. (**b**) princurve, t=125. (**c**) incremental SCMS, t=100. (**d**) incremental SCMS, t=125. (**e**) slpc, t=100. (**f**) slpc, t=125.

**Seismic data.** Seismic data spanning long periods of time are essential for a thorough understanding of earthquakes. The “Centennial Earthquake Catalog” [42] aims at providing a realistic picture of the seismicity distribution on Earth. It consists in a global catalog of locations and magnitudes of instrumentally recorded earthquakes from 1900 to 2008. We focus on a particularly representative seismic active zone (a lithospheric border close to Australia) whose longitude is between E$130^{\circ }$ to E$180^{\circ }$ and latitude between S$70^{\circ }$ to N$30^{\circ }$, with T=218 seismic recordings. As shown in Figure 10, slpc recovers nicely the tectonic plate boundary, but both princurve and incremental SCMS with well-calibrated bandwidth fail to do so.

Lastly, since no ground truth is available, we used the $R^{2}$ coefficient to assess the performance (residuals are replaced by the squared distance between data points and their projections onto the principal curve). The average over 10 trials was 0.990.

**Figure 11:**
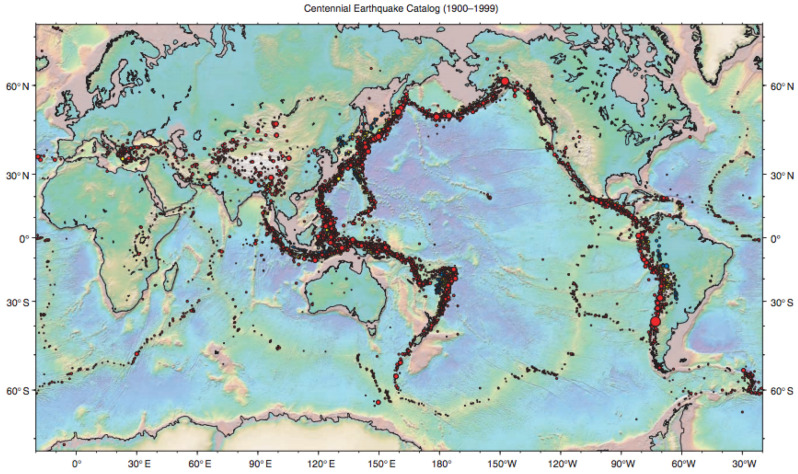
Seismic data from https://earthquake.usgs.gov/data/centennial/.

**Back to Seismic Data.**Figure 11 was taken from the USGS website (https://earthquake.usgs.gov/data/centennial/) and gives the global locations of earthquakes for the period 1900–1999. The seismic data (latitude, longitude, magnitude of earthquakes, etc.) used in the present paper may be downloaded from this website.

**Figure 12:**
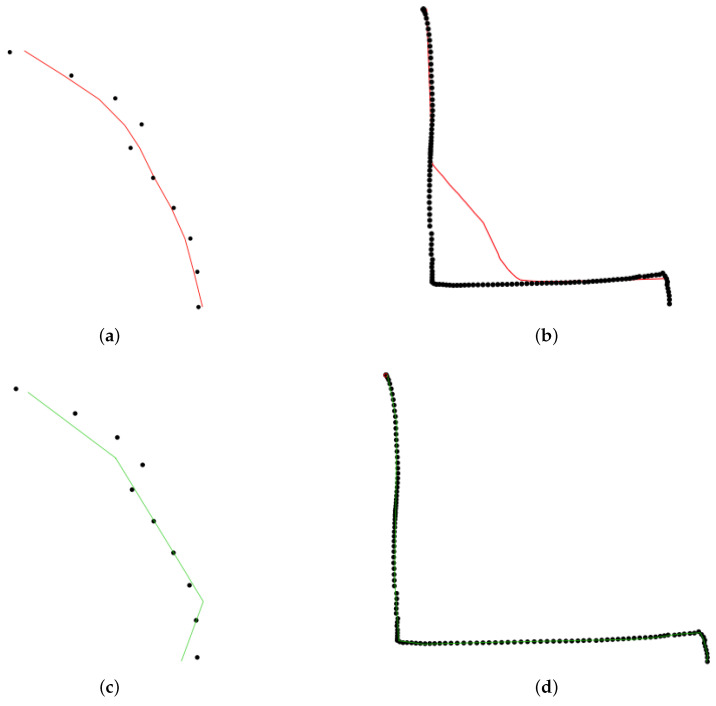
Daily commute data. Black dots represent collected locations $x_{1:t}$. The red point is the new observation $x_{t+1}$. princurve (solid red) and slpc (solid green). (**a**) t=10, princurve. (**b**) t=127, princurve. (**c**) t=10, slpc. (**d**) t=127, slpc.

**Daily Commute Data.** The identification of segments of personal daily commuting trajectories can help taxi or bus companies to optimize their fleets and increase frequencies on segments with high commuting activity. Sequential principal curves appear to be an ideal tool to address this learning problem: we tested our algorithm on trajectory data from the University of Illinois at Chicago (https://www.cs.uic.edu/~boxu/mp2p/gps_data.html). The data were obtained from the GPS reading systems carried by two of the laboratory members during their daily commute for 6 months in the Cook county and the Dupage county of Illinois. Figure 12 presents the learning curves yielded by princurve and slpc on geolocalization data for the first person, on May 30. A particularly remarkable asset of slpc is that abrupt curvature in the data sequence was perfectly captured, whereas princurve does not enjoy the same flexibility. Again, we used the $R^{2}$ coefficient to assess the performance (where residuals are replaced by the squared distances between data points and their projections onto the principal curve). The average over 10 trials was 0.998.

## 6. Proofs

This section contains the proof of Theorem 2 (note that Theorem 1 is a straightforward consequence, with $\eta_{t}=\eta$, t=0,…,T) and the proof of Theorem 3 (which involves intermediary lemmas). Let us first define for each t=0,…,T the following forecaster sequence ${\left({\hat{f}}_{t}^{⋆}\right)}_{t}$

$$\begin{array}{cc} \; & {\hat{f}}_{0}^{⋆}=\underset{f\in F_{p}}{\arg\inf}\;\left\{\Delta_{f,0}\right\}=\underset{f\in F_{p}}{\arg\inf}\left\{\frac{1}{\eta_{0}}h\left(f\right)-\frac{1}{\eta_{0}}z_{f}\right\},\\ \; & {\hat{f}}_{t}^{⋆}=\underset{f\in F_{p}}{\arg\inf}\;\left\{\sum_{s=0}^{t}\Delta_{f,s}\right\}=\underset{f\in F_{p}}{\arg\inf}\left\{\sum_{s=1}^{t}\Delta \left(f,x_{s}\right)+\frac{1}{\eta_{t-1}}h\left(f\right)-\frac{1}{\eta_{t-1}}z_{f}\right\},\;t\geq 1. \end{array}$$

Note that ${\hat{f}}_{t}^{⋆}$ is an “illegal” forecaster since it peeks into the future. In addition, denote by

$$f^{⋆}=\underset{f\in F_{p}}{\arg\inf}\left\{\sum_{t=1}^{T}\Delta \left(f,x_{t}\right)+\frac{1}{\eta_{T}}h\left(f\right)\right\}$$

the polygonal line in $F_{p}$ which minimizes the cumulative loss in the first *T* rounds plus a penalty term. $f^{⋆}$ is deterministic, and ${\hat{f}}_{t}^{⋆}$ is a random quantity (since it depends on $z_{f}$, $f\in F_{p}$ drawn from π). If several f attain the infimum, we chose $f_{T}^{⋆}$ as the one having the smallest complexity. We now enunciate the first (out of three) intermediary technical result.

**Lemma** **1.** *For any sequence $x_{1},\ldots ,x_{T}$ in $B(0,\sqrt{d}R)$,*

(5) $$\sum_{t=0}^{T}\Delta_{{\hat{f}}_{t}^{⋆},t}\leq \sum_{t=0}^{T}\Delta_{{\hat{f}}_{T}^{⋆},t},\;\pi \text{-}\mathrm{almost}\;\mathrm{surely}.$$

**Proof.**  Proof by induction on *T*. Clearly (5) holds for T=0. Assume that (5) holds for T−1:

$$\sum_{t=0}^{T-1}\Delta_{{\hat{f}}_{t}^{⋆},t}\leq \sum_{t=0}^{T-1}\Delta_{{\hat{f}}_{T-1}^{⋆},t}.$$

Adding $\Delta_{{\hat{f}}_{T}^{⋆},T}$ to both sides of the above inequality concludes the proof. □

By (5) and the definition of ${\hat{f}}_{T}^{⋆}$, for k≥1, we have π-almost surely that

$$\begin{array}{cc} \sum_{t=1}^{T}\Delta \left({\hat{f}}_{t}^{⋆},x_{t}\right) & \leq \sum_{t=1}^{T}\Delta \left({\hat{f}}_{T}^{⋆},x_{t}\right)+\frac{1}{\eta_{T}}h\left({\hat{f}}_{T}^{⋆}\right)-\frac{1}{\eta_{T}}Z_{{\hat{f}}_{T}^{⋆}}+\sum_{t=0}^{T}\left(\frac{1}{\eta_{t-1}}-\frac{1}{\eta_{t}}\right)\left(h\left({\hat{f}}_{t}^{⋆}\right)-Z_{{\hat{f}}_{t}^{⋆}}\right)\\ \; & \leq \sum_{t=1}^{T}\Delta \left(f^{⋆},x_{t}\right)+\frac{1}{\eta_{T}}h\left(f^{⋆}\right)-\frac{1}{\eta_{T}}Z_{f^{⋆}}+\sum_{t=0}^{T}\left(\frac{1}{\eta_{t-1}}-\frac{1}{\eta_{t}}\right)\left(h\left({\hat{f}}_{t}^{⋆}\right)-Z_{{\hat{f}}_{t}^{⋆}}\right)\\ \; & =\underset{f\in F_{p}}{\inf}\left\{\sum_{t=1}^{T}\Delta \left(f,x_{t}\right)+\frac{1}{\eta_{T}}h\left(f\right)\right\}-\frac{1}{\eta_{T}}Z_{f^{⋆}}+\sum_{t=0}^{T}\left(\frac{1}{\eta_{t-1}}-\frac{1}{\eta_{t}}\right)\left(h\left({\hat{f}}_{t}^{⋆}\right)-Z_{{\hat{f}}_{t}^{⋆}}\right), \end{array}$$

where $1/\eta_{-1}=0$ by convention. The second and third inequality is due to respectively the definition of ${\hat{f}}_{T}^{⋆}$ and $f_{T}^{⋆}$. Hence

$$\begin{array}{cc} E\left[\sum_{t=1}^{T}\Delta \left({\hat{f}}_{t}^{⋆},x_{t}\right)\right] & \leq \underset{f\in F_{p}}{\inf}\left\{\sum_{t=1}^{T}\Delta \left(f,x_{t}\right)+\frac{1}{\eta_{T}}h\left(f\right)\right\}-\frac{1}{\eta_{T}}E\left[Z_{f_{T}^{⋆}}\right]\\ & +\sum_{t=0}^{T}E\left[\left(\frac{1}{\eta_{t}}-\frac{1}{\eta_{t-1}}\right)\left(-h\left({\hat{f}}_{t}^{⋆}\right)+Z_{{\hat{f}}_{t}^{⋆}}\right)\right]\\ \; & \leq \underset{f\in F_{p}}{\inf}\left\{\sum_{t=1}^{T}\Delta \left(f,x_{t}\right)+\frac{1}{\eta_{T}}h\left(f\right)\right\}+\sum_{t=1}^{T}\left(\frac{1}{\eta_{t}}-\frac{1}{\eta_{t-1}}\right)E\left[\underset{f\in F_{p}}{\sup}\left(-h\left(f\right)+Z_{f}\right)\right]\\ \; & =\underset{f\in F_{p}}{\inf}\left\{\sum_{t=1}^{T}\Delta \left(f,x_{t}\right)+\frac{1}{\eta_{T}}h\left(f\right)\right\}+\frac{1}{\eta_{T}}E\left[\underset{f\in F_{p}}{\sup}\left(-h\left(f\right)+Z_{f}\right)\right], \end{array}$$

where the second inequality is due to $E[Z_{f_{T}^{⋆}}]=0$ and $\left(\frac{1}{\eta_{t}}-\frac{1}{\eta_{t-1}}\right)>0$ for t=0,1,…,T since $\eta_{t}$ is decreasing in *t* in Theorem 2. In addition, for y≥0, one has

$$P\left(-h\left(f\right)+Z_{f}>y\right)=e^{-h(f)-y}.$$

Hence, for any y≥0

$$P\left(\underset{f\in F_{p}}{\sup}\left(-h\left(f\right)+Z_{f}\right)>y\right)\leq \sum_{f\in F_{p}}P\left(Z_{f}\geq h\left(f\right)+y\right)=\sum_{f\in F_{p}}e^{-h(f)}e^{-y}=ue^{-y},$$

where $u=\sum_{f\in F_{p}}e^{-h(f)}$. Therefore, we have

$$\begin{array}{cc} E\left[\underset{f\in F_{p}}{\sup}\left(-h\left(f\right)+Z_{f}\right)-\ln u\right] & \leq E\left[\max\left(0,\underset{f\in F_{p}}{\sup}\left(-h\left(f\right)+Z_{f}-\ln u\right)\right)\right]\\ \; & \leq \int_{0}^{\infty }P\left(\max\left(0,\underset{f\in F_{p}}{\sup}\left(-h\left(f\right)+Z_{f}-\ln u\right)\right)>y\right)dy\\ \; & \leq \int_{0}^{\infty }P\left(\underset{f\in F_{p}}{\sup}\left(-h\left(f\right)+Z_{f}\right)>y+\ln u\right)dy\\ \; & \leq \int_{0}^{\infty }ue^{-(y+\ln u)}dy=1. \end{array}$$

We thus obtain

(6) $$E\left[\sum_{t=1}^{T}\Delta \left({\hat{f}}_{t}^{⋆},x_{t}\right)\right]\leq \underset{f\in F_{p}}{\inf}\left\{\sum_{t=1}^{T}\Delta \left(f,x_{t}\right)+\frac{1}{\eta_{T}}h\left(f\right)\right\}+\frac{1}{\eta_{T}}\left(1+\ln\sum_{f\in F_{p}}e^{-h(f)}\right).$$

Next, we control the regret of Algorithm 2.

**Lemma** **2.** *Assume that $z_{f}$ is sampled from the symmetric exponential distribution in R, i.e., $\pi \left(z\right)=e^{-z}1_{\left\{z>0\right\}}$. Assume that $\sup_{t=1,\ldots ,T}\eta_{t-1}\leq \frac{1}{d{\left(2R+\delta \right)}^{2}}$, and define $c_{0}=d{\left(2R+\delta \right)}^{2}$. Then for any sequence $\left(x_{t}\right)\in B\left(0,\sqrt{d}R\right)$, t=1,…,T,*

(7) $$\sum_{t=1}^{T}E\left[\Delta \left({\hat{f}}_{t},x_{t}\right)\right]\leq \sum_{t=1}^{T}\left(1+\eta_{t-1}c_{0}\left(e-1\right)\right)E\left[\Delta \left({\hat{f}}_{t}^{⋆},x_{t}\right)\right].$$

**Proof.**  Let us denote by

$$F_{t}\left(Z_{f}\right)=\Delta \left({\hat{f}}_{t},x_{t}\right)=\Delta \left(\underset{f\in F}{\arg\inf}\left(\sum_{s=1}^{t-1}\Delta \left(f,x_{s}\right)+\frac{1}{\eta_{t-1}}h\left(f\right)-\frac{1}{\eta_{t-1}}Z_{f}\right),x_{t}\right)$$

the instantaneous loss suffered by the polygonal line ${\hat{f}}_{t}$ when $x_{t}$ is obtained. We have

$$\begin{array}{cc} E[\Delta \left({\hat{f}}_{t}^{⋆},x_{t}\right)] & =\int F_{t}\left(z-\eta_{t-1}\Delta \left(f,x_{t}\right)\right)\pi \left(z\right)dz\\ & =\int F_{t}\left(z\right)\pi \left(z+\eta_{t-1}\Delta \left(f,x_{t}\right)\right)dz\\ \; & =\int F_{t}\left(z\right)e^{-\left(z+\eta_{t-1}\Delta \left(f,x_{t}\right)\right)}dz\\ \; & \geq e^{-\eta_{t-1}d{\left(2R+\delta \right)}^{2}}\int F_{t}\left(z\right)e^{-z}dz\\ & =e^{-\eta_{t-1}d{\left(2R+\delta \right)}^{2}}E\left[\Delta \left({\hat{f}}_{t},x_{t}\right)\right], \end{array}$$

where the inequality is due to the fact that $\Delta \left(f,x\right)\leq d{\left(2R+\delta \right)}^{2}$ holds uniformly for any $f\in F_{p}$ and $x\in B(0,\sqrt{d}R)$. Finally, summing on *t* on both sides and using the elementary inequality $e^{x}\leq 1+\left(e-1\right)x$ if x∈(0,1) concludes the proof. □

**Lemma** **3.** *For k∈⟦1,p⟧, we control the cardinality of set $\left\{f\in F_{p},K\left(f\right)=k\right\}$ as*

$$\begin{array}{cc} \ln\left|\left\{f\in F_{p},K\left(f\right)=k\right\}\right| & \leq \left(\ln\left(8peV_{d}\right)+3d^{\frac{3}{2}}-d\right)k+\left(\frac{\ln2}{\delta \sqrt{d}}+\frac{d}{\delta }\right)L+d\ln\left(\frac{\sqrt{d}\left(2R+\delta \right)}{\delta }\right)\\ \; & \overset{\Delta }{=}c_{1}k+c_{2}L+c_{3}, \end{array}$$

*where $V_{d}$ denotes the volume of the unit ball in $R^{d}$.*

**Proof.**  First, let $N_{k,\delta }$ denote the set of polygonal lines with *k* segments and whose vertices are in $Q_{\delta }$. Notice that $N_{k,\delta }$ is different from $\left\{f\in F_{p},K\left(f\right)=k\right\}$ and that

{f∈Fp,K(f)=k}≤pkNk,δ.

Hence

ln{f∈Fp,K(f)=k}≤lnpk+lnNk,δ≤klnpek+kln8Vd+3d32−d+ln2dδ+dδL+dlnd(2R+δ)δ≤kln(pe)+kln8Vd+3d32−d+ln2dδ+dδL+dlnd(2R+δ)δ,

where the second inequality is a consequence to the elementary inequality pk≤pekk combined with Lemma 2 in [16]. □

We now have all the ingredients to prove Theorem 1 and Theorem 2.

First, combining (6) and (7) yields that

$$\begin{array}{cc} \sum_{t=1}^{T}E\left[\Delta ({\hat{f}}_{t},x_{t})\right] & \leq \underset{f\in F_{p}}{\inf}\left\{\sum_{t=1}^{T}\Delta \left(f,x_{t}\right)+\frac{1}{\eta_{T}}h\left(f\right)\right\}+\frac{1}{\eta_{T}}\left(\frac{1}{2}+\ln\sum_{f\in F_{p}}e^{-h(f)}\right)\\ & \;\;+c_{0}\left(e-1\right)\sum_{t=1}^{T}\eta_{t-1}E\left[\Delta ({\hat{f}}_{t}^{⋆},x_{t})\right]\\ \; & \leq \underset{k\in ⟦1,p⟧}{\inf}\left\{\underset{\begin{array}{c} f\in F_{p}\\ K(f)=k \end{array}}{\inf}\left\{\sum_{t=1}^{T}\Delta \left(f,x_{t}\right)+\frac{h(f)}{\eta_{T}}\right\}\right\}+\frac{1}{\eta_{T}}\left(\frac{1}{2}+\ln\sum_{f\in F_{p}}e^{-h(f)}\right)\\ & \;\;+c_{0}\left(e-1\right)\sum_{t=1}^{T}\eta_{t-1}E\left[\Delta ({\hat{f}}_{t}^{⋆},x_{t})\right]. \end{array}$$

Assume that $\eta_{t}=\eta$, t=0,…,T and $h\left(f\right)=c_{1}K\left(f\right)+c_{2}L+c_{3}$ for $f\in F_{p}$, then $(\frac{1}{2}+\sum_{f\in F_{p}}e^{-h(f)})\leq 0$ and moreover

$$\begin{array}{cc} \sum_{t=1}^{T}E\left[\Delta ({\hat{f}}_{t},x_{t})\right] & \leq S_{T,h,\eta }+\frac{1}{\eta }\left(\frac{1}{2}+\ln\sum_{f\in F_{p}}e^{-h(f)}\right)+c_{0}\left(e-1\right)\eta \sum_{t=1}^{T}E\left[\Delta ({\hat{f}}_{t}^{⋆},x_{t})\right]\\ \; & \leq S_{T,h,\eta }+c_{0}\left(e-1\right)\eta S_{T,h,\eta }\\ \; & \leq S_{T,h,\eta }+\eta c_{0}\left(e-1\right)\underset{f\in F_{p}}{\inf}\sum_{t=1}^{T}\Delta \left(f,x_{t}\right)+c_{0}\left(e-1\right)\left(c_{1}p+c_{2}L+c_{3}\right), \end{array}$$

where

$$S_{T,h,\eta }=\underset{k\in ⟦1,p⟧}{\inf}\left\{\underset{\begin{array}{c} f\in F_{p}\\ K(f)=k \end{array}}{\inf}\left\{\sum_{t=1}^{T}\Delta \left(f,x_{t}\right)+\frac{h(f)}{\eta }\right\}\right\}$$

and the second inequality is obtained with Lemma 1. By setting

$$\eta =\sqrt{\frac{c_{1}p+c_{2}L+c_{3}}{c_{0}\left(e-1\right)\inf_{f\in F_{p}}\sum_{t=1}^{T}\Delta \left(f,x_{t}\right)}}$$

we obtain

$$\begin{array}{c} \sum_{t=1}^{T}E\left[\Delta ({\hat{f}}_{t},x_{t})\right]\leq \underset{k\in ⟦1,p⟧}{\inf}\left\{\underset{\begin{array}{c} f\in F_{p}\\ K(f)=k \end{array}}{\inf}\left\{\sum_{t=1}^{T}\Delta \left(f,x_{t}\right)+\sqrt{c_{0}\left(e-1\right)r_{T,k,L}}\right\}\right\}\\ \;\;\;\;\;\;\;\;\;\;\;\;\;\;\;\;+\sqrt{c_{0}\left(e-1\right)L_{T,p,L}}+c_{0}\left(e-1\right)c_{1}p+c_{2}L+c_{3}, \end{array}$$

where $r_{T,k,L}=\inf_{f\in F_{p}}\sum_{t=1}^{T}\Delta \left(f,x_{t}\right)\left(c_{1}k+c_{2}L+c_{3}\right)$. This proves Theorem 1.

Finally, assume that

$$\eta_{0}=\frac{\sqrt{c_{1}p+c_{2}L+c_{3}}}{c_{0}\sqrt{\left(e-1\right)}}\;\mathrm{and}\;\eta_{t}=\frac{\sqrt{c_{1}p+c_{2}L+c_{3}}}{c_{0}\sqrt{(e-1)t}},\;t=1,\ldots ,T.$$

Since $E\left[\Delta ({\hat{f}}_{t}^{⋆},x_{t})\right]\leq c_{0}$ for any t=1,…,T, we have

$$\begin{array}{cc} \sum_{t=1}^{T}E\left[\Delta ({\hat{f}}_{t},x_{t})\right] & \leq \underset{k\in ⟦1,p⟧}{\inf}\left\{\underset{\begin{array}{c} f\in F_{p}\\ K(f)=k \end{array}}{\inf}\left\{\sum_{t=1}^{T}\Delta \left(f,x_{t}\right)+\frac{h(f)}{\eta_{T}}\right\}\right\}+\frac{1}{\eta_{T}}\left(1+\ln\sum_{f\in F_{p}}e^{-h(f)}\right)\\ & \;+c_{0}^{2}\left(e-1\right)\sum_{t=1}^{T}\eta_{t-1}\\ \; & \leq \underset{k\in ⟦1,p⟧}{\inf}\left\{\underset{\begin{array}{c} f\in F_{p}\\ K(f)=k \end{array}}{\inf}\left\{\sum_{t=1}^{T}\Delta \left(f,x_{t}\right)+c_{0}\sqrt{\left(e-1\right)T\left(c_{0}k+c_{2}L+c_{3}\right)}\right\}\right\}\\ & \;\;\;\;\;\;\;\;\;+2c_{0}\sqrt{\left(e-1\right)T\left(c_{0}p+c_{2}L+c_{3}\right)}, \end{array}$$

which concludes the proof of Theorem 2.

**Lemma** **4.** *Using Algorithm 3, if 0<ϵ≤1, 0<β<1, $\alpha \geq \frac{\left(1-\beta \right)c_{0}}{\beta }$ and $\left|U\left({\hat{f}}_{t-1}\right)\right|\geq 2$ for all t≥2, where $\left|U\left({\hat{f}}_{t-1}\right)\right|$ is the cardinality of $U\left({\hat{f}}_{t-1}\right)$, then we have*

$$\sum_{t=1}^{T}E\left[r_{{\hat{f}}_{t},t}\right]\geq \sum_{t=1}^{T}E\left[{\hat{r}}_{{\hat{\sigma }}^{t}\left(A_{t}\right),t}\right]-2\left(1-\epsilon \right)\alpha \beta \sum_{t=1}^{T}\left|U\left({\hat{f}}_{t-1}\right)\right|.$$

**Proof.**  First notice that $A_{t}=U\left({\hat{f}}_{t-1}\right)$ if $I_{t}=0$, and that for t≥2

$$\begin{array}{cc} E\left[r_{{\hat{f}}_{t},t}|H_{t},I_{t}=0\right]= & E\left[r_{{\hat{\sigma }}^{t}\left(A_{t}\right),t}|H_{t},I_{t}=0\right]\\ = & \sum_{f\in A_{t}\cap cond(t)}r_{f,t}P\left({\hat{\sigma }}^{t}\left(A_{t}\right)=f\;|H_{t}\right)+\sum_{f\in A_{t}\cap {cond(t)}^{c}}r_{f,t}P\left({\hat{\sigma }}^{t}\left(A_{t}\right)=f\;|H_{t}\right)\\ \geq & \sum_{f\in A_{t}\cap cond(t)}r_{f,t}+\sum_{f\in A_{t}\cap {cond(t)}^{c}}\alpha P\left({\hat{\sigma }}^{t}\left(A_{t}\right)=f\;|H_{t}\right)\\ & -\left(1-\beta \right)\sum_{f\in A_{t}\cap cond(t)}r_{f,t}-\sum_{f\in A_{t}\cap {cond(t)}^{c}}\left(\alpha -r_{f,t}\right)P\left({\hat{\sigma }}^{t}\left(A_{t}\right)=f\;|H_{t}\right)\\ = & E\left[{\hat{r}}_{{\hat{\sigma }}^{t}\left(A_{t}\right),t}|H_{t},I_{t}=0\right]-\left(1-\beta \right)\sum_{f\in A_{t}\cap cond(t)}r_{f,t}\\ & -\sum_{f\in A_{t}\cap {cond(t)}^{c}}\left(\alpha -r_{f,t}\right)P\left({\hat{\sigma }}^{t}\left(A_{t}\right)=f\;|H_{t}\right)\\ \geq & E\left[{\hat{r}}_{{\hat{\sigma }}^{t}\left(A_{t}\right),t}|H_{t},I_{t}=0\right]-\left(1-\beta \right)c_{0}\left|A_{t}\right|-\alpha \beta \left|A_{t}\right|\\ \geq & E\left[{\hat{r}}_{{\hat{\sigma }}^{t}\left(A_{t}\right),t}|H_{t},I_{t}=0\right]-2\alpha \beta \left|A_{t}\right|, \end{array}$$

where $cond{\left(t\right)}^{c}$ denotes the complement of set cond(t). The first inequality above is due to the assumption that for all $f\in A_{t}\cap cond\left(t\right)$, we have $P\left({\hat{\sigma }}^{t}\left(A_{t}\right)=f\;|H_{t}\right)\geq \beta$. For t=1, the above inequality is trivial since ${\hat{r}}_{{\hat{\sigma }}^{1}\left(U\left({\hat{f}}_{0}\right)\right),1}\equiv 0$ by its definition. Hence, for t≥1, one has

(8) $$\begin{array}{cc} E\left[r_{{\hat{f}}_{t},t}|H_{t}\right] & =\epsilon E\left[r_{{\hat{\sigma }}^{t}\left(F_{p}\right),t}|H_{t},I_{t}=1\right]+\left(1-\epsilon \right)E\left[r_{{\hat{\sigma }}^{t}\left(A_{t}\right),t}|H_{t},I_{t}=0\right]\\ \; & \geq E\left[{\hat{r}}_{{\hat{f}}_{t},t}|H_{t}\right]-2\alpha \beta \left|A_{t}\right|. \end{array}$$

Summing on both sides of inequality (8) over *t* terminates the proof of Lemma 4. □

**Lemma** **5.** *Let ${\hat{c}}_{0}=\frac{c_{0}}{\beta }+\alpha$. If $0<\eta_{1}=\eta_{2}=\ldots =\eta_{T}=\eta <\frac{1}{{\hat{c}}_{0}}$, then we have*

$$\begin{array}{c} E\left[\max_{\hat{\sigma }}\left\{\sum_{t=1}^{T}{\hat{r}}_{\hat{\sigma }\left(A_{t}\right),t}-\frac{1}{\eta }h\left(\hat{\sigma }\left(A_{t}\right)\right)\right\}\right]-\sum_{t=1}^{T}E\left[{\hat{r}}_{{\hat{\sigma }}^{t}\left(A_{t}\right),t}\right]\leq \\ \;\;\;\;\;\;\;\;\;\;\;\;\;\;\;\;\;{\hat{c}}_{0}^{2}\left(e-1\right)\eta T+{\hat{c}}_{0}\left(e-1\right)\left(c_{1}p+c_{2}L+c_{3}\right). \end{array}$$

**Proof.**  By the definition of ${\hat{r}}_{f,t}$ in Algorithm 3, for any $f\in F_{p}$ and t≥1, we have

$${\hat{r}}_{f,t}\leq \max\left\{\frac{r_{f,t}}{P\left({\hat{f}}_{t}=f|H_{t}\right)},\alpha ,r_{f,t}\right\}\leq \max\left\{\frac{c_{0}}{\beta },\alpha \right\}\leq {\hat{c}}_{0},$$

where in the second inequality we use that $r_{f,t}\leq c_{0}$ for all f and *t*, and that $P\left({\hat{f}}_{t}=f|H_{t}\right)\geq \beta$ when $f\in U\left({\hat{f}}_{t-1}\right)\cap cond\left(t\right)$. The rest of the proof is similar to those of Lemmas 1 and 2. In fact, if we define by $\hat{\Delta }\left(f,x_{t}\right)={\hat{c}}_{0}-{\hat{r}}_{f,t}$, then one can easily observe the following relation when $I_{t}=1$ (similar relation in the case that $I_{t}$ = 0)

$$\begin{array}{cc} {\hat{f}}_{t}={\hat{\sigma }}^{t}\left(F_{p}\right) & =\underset{f\in F_{p}}{arg max}\left\{\sum_{s=1}^{t-1}{\hat{r}}_{f,s}+\frac{1}{\eta }\left(z_{f}-h\left(f\right)\right)\right\}\\ \; & =\underset{f\in F_{p}}{arg min}\left\{\sum_{s=1}^{t-1}\hat{\Delta }\left(f,x_{s}\right)+\frac{1}{\eta }\left(h\left(f\right)-z_{f}\right)\right\}. \end{array}$$

Then applying Lemmas 1 and 2 on this newly defined sequence $\hat{\Delta }\left({\hat{f}}_{t},x_{t}\right),t=1,\ldots T$ leads to the result of Lemma 5. □

The proof of the upcoming Lemma 6 requires the following submartingale inequality: let $Y_{0},\ldots Y_{T}$ be a sequence of random variable adapted to random events $H_{0},\ldots ,H_{T}$ such that for 1≤t≤T, the following three conditions hold:

$$E\left[Y_{t}|H_{t}\right]\leq 0,\;\mathrm{Var}\left(Y_{t}|H_{t}\right)\leq a^{2},\;Y_{t}-E\left[Y_{t}|H_{t}\right]\leq b.$$

Then for any λ>0,

$$P\left(\sum_{t=1}^{T}Y_{t}>Y_{0}+\lambda \right)\leq \exp\left(-\frac{\lambda^{2}}{2T(a^{2}+b^{2})}\right).$$

The proof can be found in Chung and Lu [43] (Theorem 7.3).

**Lemma** **6.** *Assume that $0<\beta <\frac{1}{\left|F_{p}\right|},\alpha \geq \frac{c_{0}}{\beta }$ and η>0, then we have*

$$\begin{array}{c} E\left[\max_{\sigma }\left\{\sum_{t=1}^{T}r_{\sigma \left(A_{t}\right),t}-\frac{1}{\eta }h\left(\sigma \left(A_{t}\right)\right)\right\}\right]-E\left[\max_{\hat{\sigma }}\left\{\sum_{t=1}^{T}{\hat{r}}_{\hat{\sigma }\left(A_{t}\right),t}-\frac{1}{\eta }h\left(\hat{\sigma }\left(A_{t}\right)\right)\right\}\right]\\ \;\;\;\;\;\;\leq \left(1-\left|F_{p}\right|\beta \right)\sqrt{2T\left[\frac{c_{0}^{2}}{\beta }+\alpha^{2}\left(1-\beta \right)+{\left(c_{0}+2\alpha \right)}^{2}\right]\ln\left(\frac{1}{\beta }\right)}+\left|F_{p}\right|\beta c_{0}T. \end{array}$$

**Proof.**  First, we have almost surely that

$$\begin{array}{cc} \max_{\sigma }\left\{\sum_{t=1}^{T}r_{\sigma \left(A_{t}\right),t}-\frac{1}{\eta }h\left(\sigma \left(A_{t}\right)\right)\right\}-\max_{\hat{\sigma }}\left\{\sum_{t=1}^{T}{\hat{r}}_{\hat{\sigma }\left(A_{t}\right),t}-\frac{1}{\eta }h\left(\hat{\sigma }\left(A_{t}\right)\right)\right\} & \leq \max_{f\in F_{p}}\sum_{t=1}^{T}\left(r_{f,t}-{\hat{r}}_{f,t}\right). \end{array}$$

Denote by $Y_{f,t}=r_{f,t}-{\hat{r}}_{f,t}$. Since

$$E\left[{\hat{r}}_{f,t}|H_{t}\right]=\left\{\begin{array}{cc} r_{f,t}+\left(1-\epsilon \right)\alpha \left(1-P\left({\hat{f}}_{t}=f|H_{t}\right)\right) & if\;f\in U\left({\hat{f}}_{t-1}\right)\cap cond\left(t\right),\\ \epsilon r_{f,t}+\left(1-\epsilon \right)\alpha & \mathrm{otherwise}, \end{array}\right.$$

and $\alpha >c_{0}\geq r_{f,t}$ uniformly for any f and *t*, we have uniformly that $E\left[Y_{t}|H_{t}\right]\leq 0$, satisfying the first condition.

For the second condition, if $f\in U\left({\hat{f}}_{t-1}\right)\cap \;cond\left(t\right)$, then

$$\begin{array}{cc} \mathrm{Var}(Y_{t}|H_{t})= & E\left[{\hat{r}}_{f,t}^{2}|H_{t}\right]-{\left(E\left[{\hat{r}}_{f,t}|H_{t}\right]\right)}^{2}\\ \leq & \epsilon r_{f,t}^{2}+\left(1-\epsilon \right)\left[\frac{r_{f,t}^{2}}{P\left({\hat{f}}_{t}=f|H_{t}\right)}+\alpha \left(1-P\left({\hat{f}}_{t}=f|H_{t}\right)\right)\right]\\ \; & -{\left[r_{f,t}+\left(1-\epsilon \right)\alpha \left(1-P\left({\hat{f}}_{t}=f|H_{t}\right)\right)\right]}^{2}\\ \leq & \frac{r_{f,t}^{2}}{\beta }+\alpha^{2}\left(1-\beta \right)\leq \frac{c_{0}^{2}}{\beta }+\alpha^{2}\left(1-\beta \right). \end{array}$$

Similarly, for $f\notin U\left({\hat{f}}_{t-1}\right)\cap cond\left(t\right)$, one can have $\mathrm{Var}\left(Y_{t}|H_{t}\right)\leq \alpha^{2}$. Moreover, for the third condition, since

$$E\left[Y_{f,t}|H_{t}\right]\geq -2\alpha ,$$

then

$$Y_{f,t}-E\left[Y_{f,t}|H_{t}\right]\leq r_{f,t}+2\alpha \leq c_{0}+2\alpha .$$

Setting $\lambda =\sqrt{2T\left[\frac{c_{0}^{2}}{\beta }+\alpha^{2}\left(1-\beta \right)+{\left(c_{0}+2\alpha \right)}^{2}\right]\ln\left(\frac{1}{\beta }\right)}$ leads to

$$P\left(\sum_{t=1}^{T}Y_{f,t}\geq \lambda \right)\leq \beta .$$

Hence the following inequality holds with probability $1-|F_{p}|\beta$

$$\max_{f\in F_{p}}\sum_{t=1}^{T}\left(r_{f,t}-{\hat{r}}_{f,t}\right)\leq \sqrt{2T\left[\frac{c_{0}^{2}}{\beta }+\alpha^{2}\left(1-\beta \right)+{\left(c_{0}+2\alpha \right)}^{2}\right]\ln\left(\frac{1}{\beta }\right)}.$$

Finally, noticing that $\max_{f\in F_{p}}\sum_{t=1}^{T}\left(r_{f,t}-{\hat{r}}_{f,t}\right)\leq c_{0}T$ almost surely, we terminate the proof of Lemma 6. □

**Proof of Theorem** **3**. Assume that p>6, $T\geq 2|F_{p}{|}^{2}$ and let

$$\begin{array}{c} \beta ={\left|F_{p}\right|}^{-\frac{1}{2}}T^{-\frac{1}{4}},\;\alpha =\frac{c_{0}}{\beta },\;{\hat{c}}_{0}=\frac{2c_{0}}{\beta },\\ \;\;\;\;\;\;\;\eta_{1}=\eta_{2}=\ldots =\eta_{T}=\frac{\sqrt{c_{1}p+c_{2}L+c_{3}}}{\sqrt{T(e-1)}{\hat{c}}_{0}},\;\epsilon =1-{\left|F_{p}\right|}^{\frac{1}{2}-\frac{3}{p}}T^{-\frac{1}{4}}. \end{array}$$

With those values, the assumptions of Lemmas 4, 5 and 6 are satisfied. Combining their results lead to the following

$$\begin{array}{cc} \sum_{t=1}^{T}E\left[r_{{\hat{f}}_{t},t}\right]\geq & \;E\left[\max_{\sigma }\left\{\sum_{t=1}^{T}r_{\sigma \left(A_{t}\right),t}-\frac{1}{\eta }h\left(\sigma \left(A_{t}\right)\right)\right\}\right]-2\alpha \beta \left(1-\epsilon \right)\sum_{t=1}^{T}\left|U\left({\hat{f}}_{t-1}\right)\right|\\ \; & -{\hat{c}}_{0}^{2}\left(e-1\right)\eta T-{\hat{c}}_{0}\left(e-1\right)\left(c_{1}p+c_{2}L+c_{3}\right)\\ \; & -\left(1-\left|F_{p}\right|\beta \right)\sqrt{2T\left[\frac{c_{0}^{2}}{\beta }+\alpha^{2}\left(1-\beta \right)+{\left(c_{0}+2\alpha \right)}^{2}\right]\ln\left(\frac{1}{\beta }\right)}-\left|F_{p}\right|\beta c_{0}T\\ \geq & E\left[\max_{\sigma }\left\{\sum_{t=1}^{T}r_{\sigma \left(A_{t}\right),t}-\frac{1}{\eta }h\left(\sigma \left(A_{t}\right)\right)\right\}\right]-\left(1-\epsilon \right){\left|F_{p}\right|}^{\frac{3}{p}}c_{0}T\\ & -{\hat{c}}_{0}^{2}\left(e-1\right)\eta T-{\hat{c}}_{0}\left(e-1\right)\left(c_{1}p+c_{2}L+c_{3}\right)\\ \; & -\left(1-\left|F_{p}\right|\beta \right)\sqrt{2T\left[\frac{c_{0}^{2}}{\beta }+\alpha^{2}\left(1-\beta \right)+{\left(c_{0}+2\alpha \right)}^{2}\right]\ln\left(\frac{1}{\beta }\right)}-\left|F_{p}\right|\beta c_{0}T\\ \geq & E\left[\max_{\sigma }\left\{\sum_{t=1}^{T}r_{\sigma \left(A_{t}\right),t}-\frac{1}{\eta }h\left(\sigma \left(A_{t}\right)\right)\right\}\right]-O\left({\left|F_{p}\right|}^{\frac{1}{2}}T^{\frac{3}{4}}\right), \end{array}$$

where the second inequality is due to the fact that the cardinality $\left|U\left({\hat{f}}_{t-1}\right)\right|$ is upper bounded by ${\left|F_{p}\right|}^{\frac{3}{p}}$ for t≥1. In addition, using the definition of $r_{f,t}$ that $r_{f,t}=c_{0}-\Delta \left(f,x_{t}\right)$ terminates the proof of Theorem 3. □

## Figures and Tables

**Table 1:** The first line is the regret (cumulative loss) on synthetic data (average over 10 trials, with standard deviation in brackets). Second and third lines are the average computation time for two values of the time horizon *T*. princurve and incremental SCMS are deterministic, hence the zero standard deviation for regret.

Ground Truth	Princurve	Incremental SCMS	slpc
2.48 (0)	26.02 (0)	19.09 (0)	20.83 (3.23)
T = 500	0.029 s (0.0001 s)	18.79 s (0.007 s)	1.44 s (0.030 s)
T = 5000	0.35 s (0.006 s)	>60 s (NA)	4.13 s (0.807 s)

**Table 2:** Regret (cumulative loss) on synthetic high dimensional data in (average over 10 trials, with standard deviation in brackets). princurve and incremental SCMS are deterministic, hence the zero standard deviation.

*Ground Truth*	Princurve	Incremental SCMS	slpc
3.290 (0)	14.204 (0)	5.38 (0)	6.797 (0.409)

## References

[1] Pearson K. (1901). On lines and planes of closest fit to systems of point in space. Philos. Mag..

[2] Spearman C. (1904). “General Intelligence”, Objectively Determined and Measured. Am. J. Psychol..

[3] Hotelling H. (1933). Analysis of a complex of statistical variables into principal components. J. Educ. Psychol..

[4] Friedsam H., Oren W.A. The application of the principal curve analysis technique to smooth beamlines. Proceedings of the 1st International Workshop on Accelerator Alignment.

[5] Brunsdon C. Path estimation from GPS tracks. Proceedings of the 9th International Conference on GeoComputation.

[6] Reinhard K., Niranjan M. (1999). Parametric Subspace Modeling Of Speech Transitions. Speech Commun..

[7] Kégl B., Krzyżak A. (2002). Piecewise linear skeletonization using principal curves. IEEE Trans. Pattern Anal. Mach. Intell..

[8] Banfield J.D., Raftery A.E. (1992). Ice floe identification in satellite images using mathematical morphology and clustering about principal curves. J. Am. Stat. Assoc..

[9] Stanford D.C., Raftery A.E. (2000). Finding curvilinear features in spatial point patterns: Principal curve clustering with noise. IEEE Trans. Pattern Anal. Mach. Intell..

[10] Hastie T., Stuetzle W. (1989). Principal curves. J. Am. Stat. Assoc..

[11] Delicado P. (2001). Another Look at Principal Curves and Surfaces. J. Multivar. Anal..

[12] Einbeck J., Tutz G., Evers L. (2005). Local principal curves. Stat. Comput..

[13] Einbeck J., Tutz G., Evers L. (2010). Data Compression and Regression through Local Principal Curves and Surfaces. Int. J. Neural Syst..

[14] Malo J., Gutiérrez J. (2006). V1 non-linear properties emerge from local-to-global non-linear ICA. Netw. Comput. Neural Syst..

[15] Ozertem U., Erdogmus D. (2011). Locally Defined Principal Curves and Surfaces. J. Mach. Learn. Res..

[16] Kégl B. (1999). Principal Curves: Learning, Design, and Applications. Ph.D. Thesis.

[17] Kégl B., Krzyżak A., Linder T., Zeger K. (2000). Learning and design of principal curves. IEEE Trans. Pattern Anal. Mach. Intell..

[18] Biau G., Fischer A. (2012). Parameter selection for principal curves. IEEE Trans. Inf. Theory.

[19] Barron A., Birgé L., Massart P. (1999). Risk bounds for model selection via penalization. Probab. Theory Relat. Fields.

[20] Birgé L., Massart P. (2007). Minimal penalties for Gaussian model selection. Probab. Theory Relat. Fields.

[21] Sandilya S., Kulkarni S.R. (2002). Principal curves with bounded turn. IEEE Trans. Inf. Theory.

[22] Cesa-Bianchi N., Lugosi G. (2006). Prediction, Learning and Games.

[23] Rudzicz F., Ghassabeh Y.A. (2015). Incremental algorithm for finding principal curves. IET Signal Process..

[24] Laparra V., Malo J. (2016). Sequential Principal Curves Analysis. arXiv.

[25] Laparra V., Jiménez S., Camps-Valls G., Malo J. (2012). Nonlinearities and Adaptation of Color Vision from Sequential Principal Curves Analysis. Neural Comput..

[26] Laparra V., Malo J. (2015). Visual Aftereffects and Sensory Nonlinearities from a single Statistical Framework. Front. Hum. Neurosci..

[27] Laparra V., Jiménez S., Tuia D., Camps-Valls G., Malo J. (2014). Principal Polynomial Analysis. Int. J. Neural Syst..

[28] Laparra V., Malo J., Camps-Valls G. (2015). Dimensionality Reduction via Regression in Hyperspectral Imagery. IEEE J. Sel. Top. Signal Process..

[29] Shawe-Taylor J., Williamson R.C. A PAC analysis of a Bayes estimator. Proceedings of the 10th annual conference on Computational Learning Theory.

[30] McAllester D.A. (1999). Some PAC-Bayesian Theorems. Mach. Learn..

[31] McAllester D.A. PAC-Bayesian Model Averaging. Proceedings of the 12th Annual Conference on Computational Learning Theory.

[32] Li L., Guedj B., Loustau S. (2018). A quasi-Bayesian perspective to online clustering. Electron. J. Stat..

[33] Guedj B. A Primer on PAC-Bayesian Learning. Proceedings of the Second Congress of the French Mathematical Society.

[34] Alquier P. (2021). User-friendly introduction to PAC-Bayes bounds. arXiv.

[35] Audibert J.Y. (2009). Fast Learning Rates in Statistical Inference through Aggregation. Ann. Stat..

[36] Hutter M., Poland J. (2005). Adaptive Online Prediction by Following the Perturbed Leader. J. Mach. Learn. Res..

[37] Auer P., Cesa-Bianchi N., Freund Y., Schapire R.E. (2003). The Nonstochastic multiarmed Bandit problem. SIAM J. Comput..

[38] Kleinberg R.D., Niculescu-Mizil A., Sharma Y. (2008). Regret Bounds for Sleeping Experts and Bandits. COLT.

[39] Kanade V., McMahan B., Bryan B. (2009). Sleeping Experts and Bandits with Stochastic Action Availability and Adversarial Rewards. Artif. Intell. Stat..

[40] Cesa-Bianchi N., Lugosi G., Stoltz G. (2005). Minimizing regret with label-efficient prediction. IEEE Trans. Inf. Theory.

[41] Neu G., Bartók G. (2013). An Efficient Algorithm for Learning with Semi-Bandit Feedback.

[42] Engdahl E.R., Villaseñor A. (2002). 41 Global seismicity: 1900–1999. Int. Geophys..

[43] Chung F., Lu L. (2006). Concentration Inequalities and Martingale Inequalities: A Survey. Internet Math..

